# Modulation of hepatic amyloid precursor protein and lipoprotein receptor-related protein 1 by chronic alcohol intake: Potential link between liver steatosis and amyloid-β

**DOI:** 10.3389/fphys.2022.930402

**Published:** 2022-09-15

**Authors:** Jerome Garcia, Rudy Chang, Ross A. Steinberg, Aldo Arce, Joshua Yang, Peter Van Der Eb, Tamara Abdullah, Devaraj V. Chandrashekar, Sydney M. Eck, Pablo Meza, Zhang-Xu Liu, Enrique Cadenas, David H. Cribbs, Neil Kaplowitz, Rachita K. Sumbria, Derick Han

**Affiliations:** ^1^ Department of Biology, University of La Verne, Verne, CA, United States; ^2^ Department of Biomedical and Pharmaceutical Sciences, School of Pharmacy, Chapman University, Irvine, CA, United States; ^3^ School of Pharmacy and Health Sciences, Keck Graduate Institute, Claremont, CA, United States; ^4^ Department of Molecular Microbiology and Immunology, USC/Norris Comprehensive Cancer Center, Keck School of Medicine, University of Southern California, Los Angeles, CA, United States; ^5^ Department of Pharmacology and Pharmaceutical Sciences, School of Pharmacy, University of Southern California, Los Angeles, CA, United States; ^6^ Institute for Memory Impairments and Neurological Disorders, University of California, Irvine, Irvine, CA, United States; ^7^ University of Southern California Research Center for Liver Diseases and Southern California Research Center for ALPD, Keck School of Medicine, University of Southern California, Los Angeles, CA, United States; ^8^ Department of Neurology, University of California, Irvine, Irvine, CA, United States

**Keywords:** amyloid precursor protein, lipoprotein receptor-related protein 1, liver, alcohol, Alzheimer’s disease, beta-amyloid

## Abstract

Heavy alcohol consumption is a known risk factor for various forms of dementia and the development of Alzheimer’s disease (AD). In this work, we investigated how intragastric alcohol feeding may alter the liver-to-brain axis to induce and/or promote AD pathology. Four weeks of intragastric alcohol feeding to mice, which causes significant fatty liver (steatosis) and liver injury, caused no changes in AD pathology markers in the brain [amyloid precursor protein (APP), presenilin], except for a decrease in microglial cell number in the cortex of the brain. Interestingly, the decline in microglial numbers correlated with serum alanine transaminase (ALT) levels, suggesting a potential link between liver injury and microglial loss in the brain. Intragastric alcohol feeding significantly affected two hepatic proteins important in amyloid-beta (Aβ) processing by the liver: 1) alcohol feeding downregulated lipoprotein receptor-related protein 1 (LRP1, ∼46%), the major receptor in the liver that removes Aβ from blood and peripheral organs, and 2) alcohol significantly upregulated APP (∼2-fold), a potentially important source of Aβ in the periphery and brain. The decrease in hepatic LRP1 and increase in hepatic APP likely switches the liver from being a remover or low producer of Aβ to an important source of Aβ in the periphery, which can impact the brain. The downregulation of LRP1 and upregulation of APP in the liver was observed in the first week of intragastric alcohol feeding, and also occurred in other alcohol feeding models (NIAAA binge alcohol model and intragastric alcohol feeding to rats). Modulation of hepatic LRP1 and APP does not seem alcohol-specific, as ob/ob mice with significant steatosis also had declines in LRP1 and increases in APP expression in the liver. These findings suggest that liver steatosis rather than alcohol-induced liver injury is likely responsible for regulation of hepatic LRP1 and APP. Both obesity and alcohol intake have been linked to AD and our data suggests that liver steatosis associated with these two conditions modulates hepatic LRP1 and APP to disrupt Aβ processing by the liver to promote AD.

## Introduction

Heavy alcohol consumption is a known risk factor for various forms of dementia and the development of Alzheimer’s disease (AD) (Fratiglioni et al., 1993; Heymann et al., 2016). Alcohol consumption may be an important modifiable risk factor for AD, but the underlying factors linking alcohol consumption and AD have not been conclusively identified. Most studies have largely focused on the direct action of alcohol on the brain, and have identified some alcohol-induced changes that may contribute to AD. Alcoholism is associated with atrophy or loss of volume in various areas of the brain including the cortex and hippocampus (De La Monte et al., 1988; Sullivan et al., 1995; Mann et al., 2001). Chronic alcohol feeding (oral alcohol diet) to mice has been shown to increase the expression of amyloid precursor protein (APP) and β-site amyloid precursor protein cleaving enzyme (BACE) in select areas of the brain including the cerebellum, hippocampus, and striatum (Kim et al., 2011). Alcohol feeding to double transgenic Alzheimer’s mice (APP23/PS45) has also been shown to increase APP expression in the brain (Huang et al., 2018). Finally, binge alcohol exposure (2–4 days binge) in rats has been shown to cause microglial dystrophy and decrease their number in the hippocampus and perirhinal and entorhinal cortices (Marshall et al., 2020). Taken together, these data suggests that alcohol consumption can have various effects on the brain that may promote AD.

While alcohol has many effects on the brain, chronic alcohol consumption most directly damages the liver, which manifests as alcoholic steatosis (fatty liver), hepatitis (inflammation plus fatty liver), and cirrhosis (fibrosis due to significant hepatocyte death). The liver performs many functions that are essential for brain health, including: metabolic homeostasis, secretion of key plasma proteins, and clearance of waste or excess biological products such as amyloid-β (Aβ) (Ghiso et al., 2004; Wang et al., 2017). This liver-brain axis is vital to brain health, and if alcohol-induced liver injury disrupts this axis, the brain may become more susceptible to AD pathology. This may be true regarding Aβ homeostasis, where the liver is involved in Aβ clearance from blood and peripheral organs (Ghiso et al., 2004; Tamaki et al., 2006; Wang et al., 2017; Bassendine et al., 2020), but is also a potential source for Aβ in the periphery and blood, which can ultimately accumulate in the brain (Sutcliffe et al., 2011; Lam et al., 2021). The liver contains several receptors that have been shown to bind to Aβ including lipoprotein receptor-related protein 1 (LRP1), receptor for advanced glycation end products (RAGE), and P-glycoprotein (Mohamed and Kaddoumi, 2013; Yamagishi and Matsui, 2015). LRP1 appears to be the most important receptor for peripheral Aβ clearance in the liver (Zlokovic et al., 2010), as suppression of hepatic LRP1 causes a 64% decline in Aβ uptake by the liver (Tamaki et al., 2006). Since Aβ can be transported across the blood-brain barrier by RAGE, hepatic LRP1 levels have been shown to strongly affect plasma- and brain-Aβ levels (Zlokovic et al., 2010; Sehgal et al., 2012). On the other hand, the liver, like most organs, contains APP, which can form Aβ that is released into circulation following the action of secretases such as presenilin 2 (PSE2). A mutated form of PSE2 that promotes AD has been shown to be heritable in the liver, but not in the brain of mice (Sutcliffe et al., 2011). A liver specific overexpression of human APP in mice has been shown to increase Aβ levels in the periphery and the brain, as well as promote AD pathology (Lam et al., 2021). Thus, key hepatic proteins such as LRP1, PSE2, and APP may determine Aβ processing and homeostasis in the liver, which profoundly affects Aβ levels in the periphery and brain.

In this study, we examined the effect of alcohol on proteins involved in the pathogenesis of AD in both the brain and liver using different alcohol feeding models, including the intragastric alcohol feeding model (Han et al., 2012; Han et al., 2017). While several studies have examined the effects of alcohol feeding on AD pathology in the brain, most of these studies have relied on oral alcohol feeding models, which only cause modest liver injury, and have not examined changes in the liver that may promote AD pathology. This work focuses on whether a liver-to-brain axis may play a significant role in alcohol-dependent AD.

## Materials and methods

All animal studies were performed under protocols approved by the University of Southern California or Chapman University, Institutional Animal Care and Use Committees.

### Animals

#### Intragastric alcohol feeding to mice and rats

Male C57BL/6J mice (∼8 weeks of age) were obtained from Jackson Laboratory (Bar Harbor, ME), whereas Wistar rats (150 g, ∼6 weeks) were obtained from Charles River (Wilmington, MA). The animals were housed in a temperature-controlled room and were acclimatized for a minimum of 3 days prior to use in experiments. Intragastric alcohol feeding to mice and rats was performed by the Southern California Research Center for ALPD and Cirrhosis. Mice and rats were implanted with a long-term gastrostomy catheter for alcohol infusion or control diet as previously described (Han et al., 2012; Han et al., 2017). Briefly, after 1 week of acclimatization, mice and rats were infused with a control diet with or without alcohol. Alcohol infusion was initiated at a dose of 22.7 g/kg/day, and gradually increased. Alcohol accounted for ∼32.9% of total caloric intake after 1 week of intragastric alcohol feeding. By 4 weeks of intragastric alcohol feeding, the caloric intake of alcohol accounted for ∼38.4% of total caloric intake. Mice were fed alcohol intragastrically for 4 weeks, while rats were fed intragastrically for 6 weeks. All animals received care according to the methods approved under institutional guidelines for the care and use of laboratory animals in research. Blood was obtained after mice were anesthetized at the indicated time periods, and serum alanine transaminase (ALT) was assessed using a kit from MilliporeSigma (St. Louis, MO, United States). Since intragastric alcohol feeding requires mice to be attached to a catheter (limiting their mobility) and are regularly intoxicated, AD cognitive test could not be performed in this model.

#### NIAAA binge alcohol feeding to mice

Male C57BL/6J mice (∼8 weeks of age) were given alcohol *via* the NIAAA binge model as previously described (Bertola et al., 2013). Briefly, mice were pair fed liquid alcohol diet (*n* = 6) or control liquid diet [maltose dextrin—Bio-Serv (Flemington, NJ, United States)] (*n* = 4) for 10 days. On the 11th day, mice were given a bolus dose of 31.5% ethanol solution (vol/vol) or 45% maltose dextrin solution (wt/vol) by oral gavage. Mice were anesthetized with a lethal dose of Euthasol (150 mg/kg, IP) ∼ 9 h following the bolus dose of alcohol, and serum was collected for ALT measurements. Cardiac perfusion was performed using ice-cold phosphate buffer saline (PBS). Brains were harvested, and hemi-brains were fixed in 4% paraformaldehyde (PFA) for immunostaining or were frozen in dry ice for Western blotting.

#### Ob/ob mice

Ob/ob, C57BL/6J, and C57BL/6NJ mice were obtained from Jackson Laboratory (Bar Harbor, ME, United States) at 6 weeks of age. All mice were fed standard chow (*ad libitum*) for up to 20 weeks (*n* = 5–6). The mice received care according to methods approved under institutional guidelines for the care and use of laboratory animals in research. C57BL/6J mice (6J mice), background for ob/ob mice also harbor another mutation that inactivates nicotinamide nucleotide transhydrogenase (NNT), a mitochondrial protein on the inner membrane responsible for transferring reducing equivalents from NADH to NADPH (Garcia et al., 2018). C57BL/6NJ mice (6NJ mice), contain functional NNT proteins and were used as an additional control for ob/ob mice.

### Brain cryosectioning

For the intragastric- and NIAAA-binge-alcohol feeding models, the right-cerebral hemi-brain of each mouse was immersion-fixed with 4% PFA in PBS for 24 h, followed by serial incubation in 10%, 20%, and 30% sucrose solution at 4°C for 24 h each, and subsequently frozen. The frozen brain tissues were mounted in Tissue-Tek OCT compound (Fisher Scientific, MA, United States), and sliced into 20 μm-thick sagittal sections at −25°C using a cryostat (Micron Instruments, CA, United States). Three sections (600 µm apart) per mouse were used for immunostaining as described below (Ou et al., 2021).

### Iba-1 immunostaining and quantification

Free-floating sagittal brain sections were washed three times for 2 min in PBS and blocked with 0.5% bovine serum albumin (BSA) in PBS containing 0.3% Triton X-100 (TX100) for 60 min at room temperature. Tissue sections were incubated with 0.5 μg/ml anti-Iba-1 rabbit antibody (to detect microgliosis) in PBS containing 0.3% TX100 and 0.5% BSA overnight at 4°C. After washing, the tissue sections were incubated in the dark with 0.1% AlexaFluor 488 donkey anti-rabbit IgG (Biolegend; CA, United States) in PBS containing 0.3% TX-100 and 0.5% BSA for 2 h at room temperature, washed, and mounted onto glass slides, coverslipped with Vectamount aqueous mounting media (Vector Laboratories, CA, United States), and sealed with nail polish. Slides were stored at 4°C until imaging. The fluorescent staining in the cortex and the hippocampus was analyzed using a Leica TCS SP5 Confocal Microscope (Leica, NJ, United States), by imaging two regions in the cortex and one region in the hippocampus. The number of microglia were manually counted in each image using the NIH ImageJ (version 1.53e, MD, United States) for microglia number quantification. For each brain region, the microglia counts were averaged to give the average number of microglia per region per mouse. The overall number of microglia represents the average of the microglia in the cortex and hippocampus. All NIH ImageJ analysis was performed by two readers blinded to the experimental groups.

### Histological analysis of liver

Livers were removed, fixed with 10% buffered formalin, embedded in paraffin, and cut into 5-μm thick sections. All specimens were stained with hematoxylin/eosin (Η&Ε) by the Histology Core at the USC Center for Liver Diseases and were evaluated under a light microscope.

### Brain homogenization and mouse Aβ (1–42) ELISA

The frozen left cerebral hemisphere without the cerebellum was pulverized to a fine homogeneous powder on dry ice, which was homogenized in five volumes of TPER with Roche complete EDTA-free Mini protease inhibitor by a mechanical homogenizer (Waverly, IA) and rotated at 4°C for 1 h. The homogenate was centrifuged at 14,000 × g for 20 min at 4°C and the supernatant was aliquoted and stored at −80°C for ELISA or immunoblotting. An aliquot of the supernatant was set aside to measure protein concentration using a bicinchoninic acid kit (Pierce Chemical Co., Rockford, IL). A part of the brain and liver homogenate supernatants and plasma samples were used for the detection of mouse Aβ (1–42) using a sandwich ELISA kit no. KMB3441 (Thermo Fisher Scientific, Waltham, MA). Absorbance (OD) was measured at 450 nm. Standard curves were fit to a four-parameter logistic regression curve and the tissue Aβ (1–42) levels were calculated and normalized based on the total protein amount in the tissue samples.

### Immunoblotting

For immunoblotting, livers were homogenized in RIPA buffer (Thermo Scientific #89900) containing protease/phosphatase inhibitor cocktail (Cell Signaling Technology, Danvers, MA, cat #5872). Protein electrophoresis occurred on 8%–12% SDS polyacrylamide gels (Biorad, Hercules, CA). Subsequently, proteins were transferred to nitrocellulose or PVDF membranes and blots were blocked with 5% (w/v) nonfat milk dissolved in Tris-buffered saline (TBS) with Tween-20. Antibodies to LRP1 (cat #64099), APP (cat #76600), Presenilin 1 (cat #5643), Presenilin 2 (cat #9979), GSK (cat #5676), and actin (cat #3700) were obtained from Cell Signaling Technology. The antibody to synaptophysin (SYP; sc-17750) was obtained from Santa Cruz Biotechnology (Dallas, Texas). Densitometry was performed using ImageJ program from NIH and normalized with actin as loading controls.

### Real-time quantitative PCR

LRP1, APP, and actin mRNA levels in the liver were assessed by RT-PCR (Table 1). RNA was extracted from liver following homogenization using column based isolation (PureLink RNA Mini Kit, Invitrogen). RNA was quantified using Tecan NanoQuant Infinite 200 Pro, and matched to a concentration of 50 ng of RNA. cDNA was then reverse transcribed using Invitrogen SuperScript III First-Strand Synthesis SuperMix (utilizing Oligo (dT)) and thermocycler. cDNA was subsequently diluted to 1:10 with RNase-free water. For RT-PCR, 2 µL of this dilution was used per well in conjunction with 12.5 µL SYBR Green (Applied Biosystems PowerSYBR Green PCR Master Mix), 9.5 µL RNase-free water, and 1 µL of mixed forward and reverse primer (Integrated DNA Technologies - Coralville, Iowa) at a concentration of 10 μM, for a total of 25 µL per well. Non-template controls were used to test for primer contamination and/or primer-dimer interactions. To assess primer validity, a standard curve was conducted to ensure efficiency percentages were between 90% and 110%. Samples were loaded into a 96 well plate with each condition run in triplicate. A Roche Lightcycler 96 was set for a preincubation cycle of 10 min at 95°C, followed by 40 cycles of 2-step amplification at 95°C for 15 s and 60°C for 60 s, and finally a melting curve of 95°C for 15 s, 65°C for 15 s, and a ramp towards 95°C increasing at a speed of 0.2°C per second. The resulting Cq data was collected from Roche’s Lightcycler 96 SW 1.1 software and was averaged and analyzed using the 2^−ΔΔCT^ method. In order to assess differences imposed by ethanol consumption on LRP1 and APP transcription, we reverse transcribed RNA isolated from liver homogenate, and conducted RT-PCR on the resultant cDNA. Interestingly, several standard genes tested as controls displayed some degree of variation between ethanol and control groups (including GAPDH, HPRT1, SRSF4, and TBP), with β-actin appearing most stable. Consequently, β-actin was chosen as control.

**TABLE 1:** List of primer sequences used in study.

Primer	Direction	Sequence
β-actin	Forward	CAT TGC TGA CAG GAT GCA GAA GG
	Reverse	TGC TGG AAG GTG GAC AGT GAG G
LRP1	Forward	CGA GAG CCT TTG TGC TGG ATG A
	Reverse	CGG ATG TCC TTC TCA ATG AGG G
LRP1-v2	Forward	GAC GTG CAA AGA TTT TGA CGA G
	Reverse	AGC AGG TAG CCT TCA ACA CAG
APP	Forward	TCC GTG TGA TCT ACG AGC GCA T
	Reverse	GCC AAG ACA TCG TCG GAG TAG G

### Statistical analysis

Numerical data is presented as mean ± SD, and all the statistical analysis was performed using GraphPad Prism 9 (GraphPad Software Inc., CA). Two-sample t test or ANOVA with repeated measures were performed when appropriate. Correlation analysis was performed using the Pearson correlation r. A two-tailed *p* < 0.05 was defined as statistically significant.

## Results

### Effect of intragastric alcohol feeding on AD-relevant alterations in the brains of mice

**FIGURE 1:**
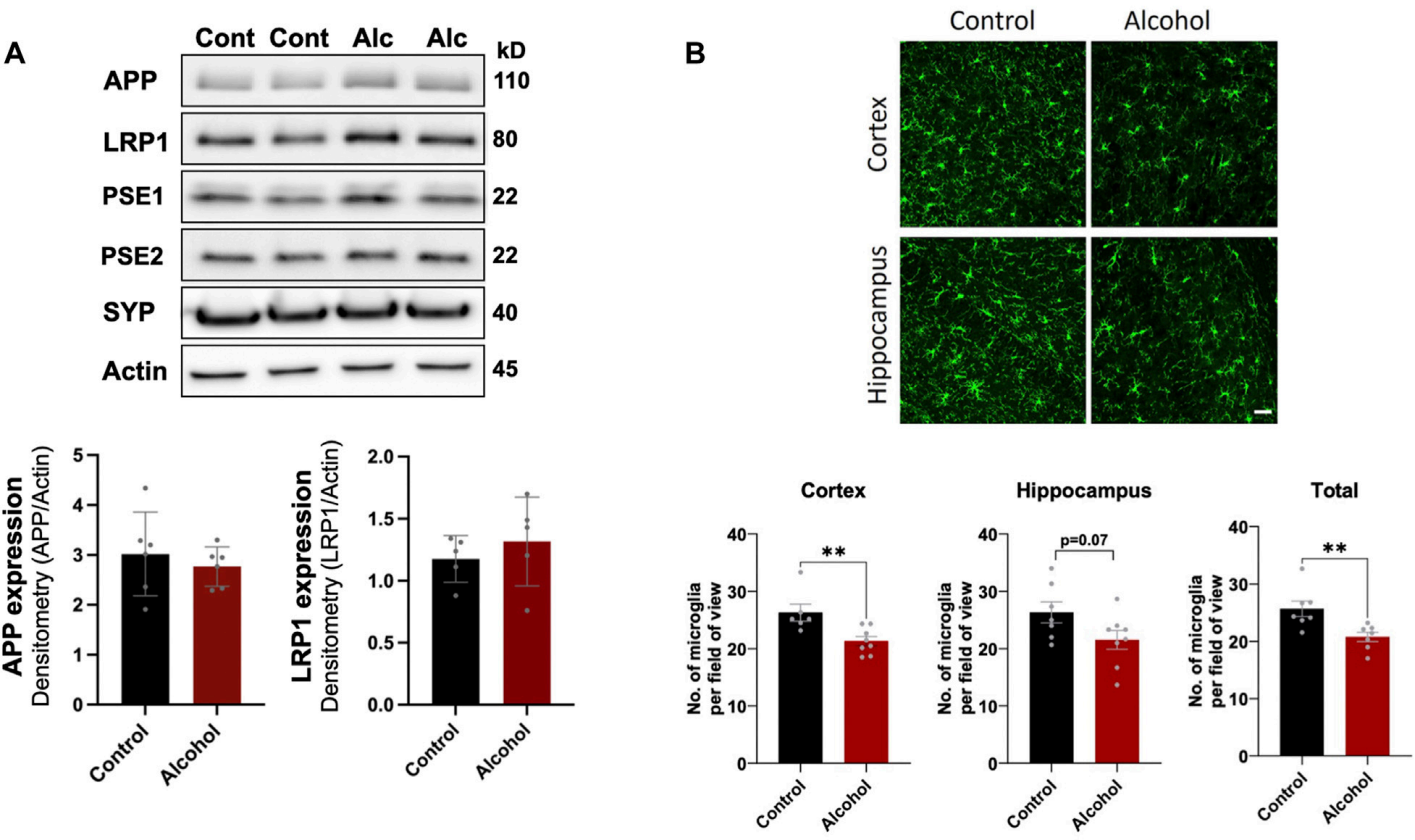
Effect of intragastric alcohol feeding on AD-relevant alterations in the brains of mice. **(A)** Expression of APP, PSE1, PSE2, LRP1, and synaptophysin (SYN), proteins that are involved in Aβ synthesis, transport, and neuronal health, respectively, following 4 weeks of intragastric alcohol feeding to mice. All immunoblots shown are representative samples from five experiments. Densitometry for APP and LRP1 in the brain was performed using NIH ImageJ. β-actin was used as the loading control. **(B)** Microglial numbers (Iba-1-stained cells) in the brain following 4 weeks of intragastric alcohol feeding. The images show Iba-1-stained cells in the cortex and hippocampus (scale bar represents 50 µm). Each Iba-1-stained image was manually read to count the number of microglia per image using NIH ImageJ. For each brain region, the microglia counts were averaged to give the average number of microglia per region per mouse. *n* = 6–7 mice per group. Results are mean ± SD. **p < 0.01. Original blots are presented in Supplementary Figures.

We first examined the effect intragastric alcohol feeding had on important proteins involved in Aβ production and transport, synaptic health, and microgliosis, which are dysregulated in AD brains (Crews and Masliah, 2010). No significant changes were observed in the protein levels of APP, LRP1, PSE1, PSE2, and synaptophysin (SYP), a synaptic protein that has been reported to decline in AD (Figure 1A). However, interestingly 4 weeks of intragastric alcohol feeding was associated with a significant decline in number of microglial cells (Iba-1stained cells) in the cortex (*p* < 0.01) and in the cortex and hippocampus combined (*p* < 0.01) (Figure 1B). Microglial cells in the hippocampus also experience a decline, but that decline was not statistically significant.

### Effect of intragastric alcohol feeding on AD-relevant alterations in the livers of mice

**FIGURE 2:**
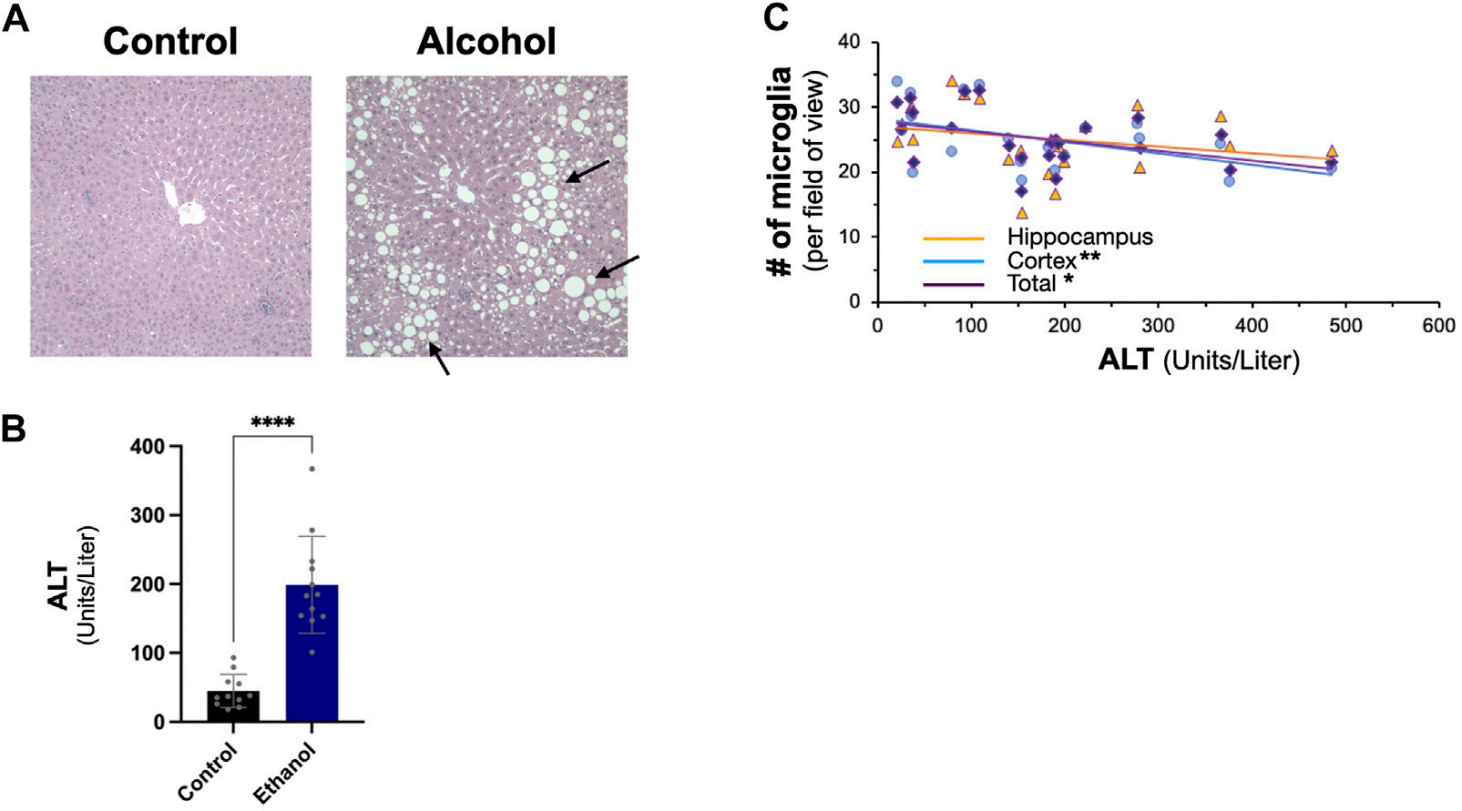
Effect of intragastric alcohol feeding on liver injury and its relationship with microglial number in the brain. **(A)** Hematoxylin and eosin (H&E) histology of mice liver following 4 weeks of intragastric alcohol feeding (×200 magnification). The arrows point to lipid droplets in the liver associated with steatosis. Representative samples of intragastric alcohol fed and control mice are shown. **(B)** ALT levels in serum following 4 weeks of intragastric alcohol feeding to mice. *n* = 6–7 mice. Results are mean ± SD. **(C)** Scatter plot showing the relationship between liver injury (ALT levels) and microglial number in the brain. ****p < 0.0001, **p < 0.01, or *p < 0.05 vs. control.

We next examined the effect of intragastric alcohol feeding on important proteins involved in Aβ production and transport in the liver, the major site of alcohol-induced injury. In agreement with our previously published results, 4 weeks of intragastric alcohol feeding caused extensive fatty liver (steatosis) (Figure 2A) and significantly increased ALT levels (marker of hepatocyte death) in the plasma (Figure 2B). A possible connection between liver injury and the brain was observed, as ALT levels were negatively correlated with microglial numbers in the cortex (Pearson r = −0.58, *p* < 0.01) and cortex and hippocampus combined (Pearson r = −0.50, *p* < 0.05). The greater the liver injury, the greater the decline in microglial numbers with alcohol feeding was observed (Figure 2C).

**FIGURE 3:**
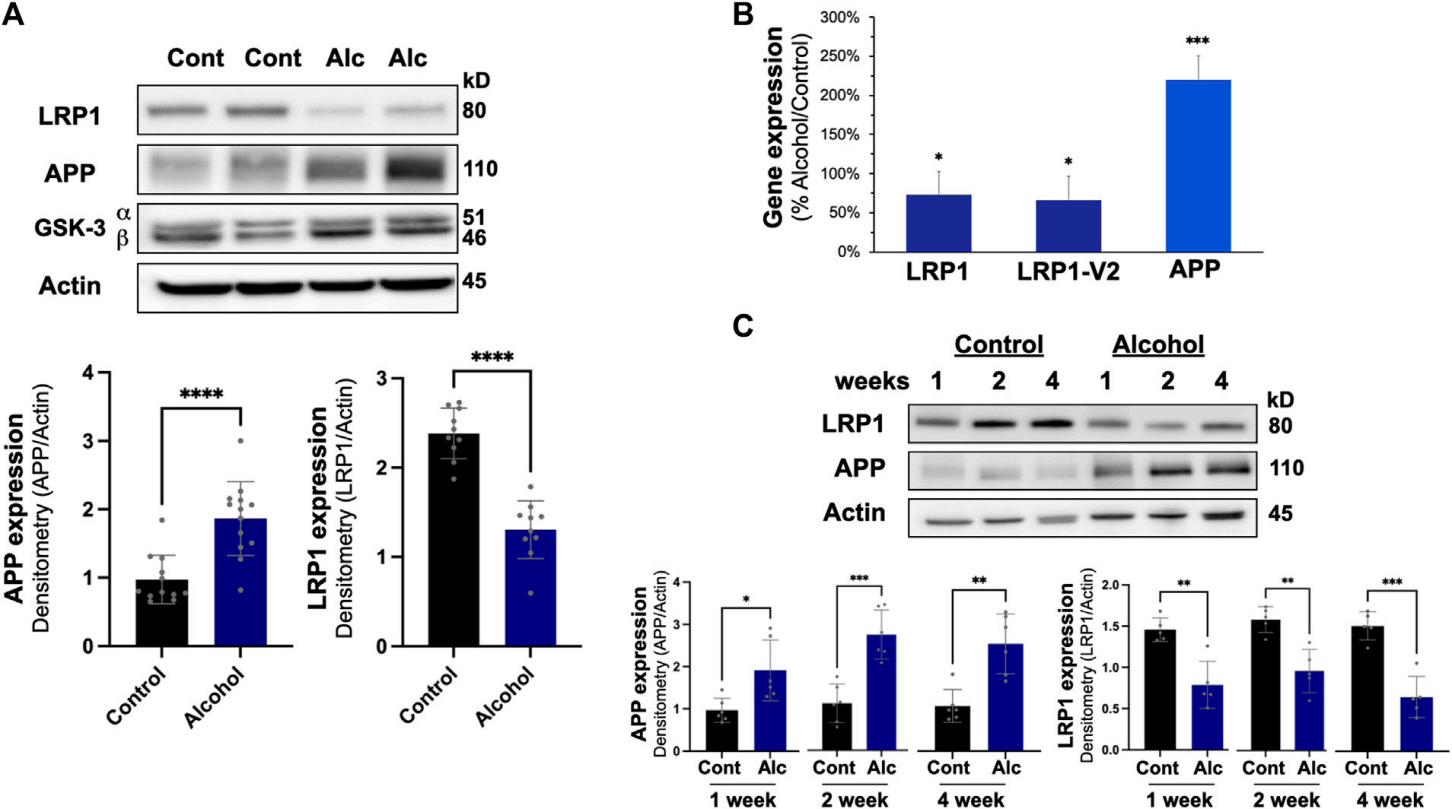
Effect of intragastric alcohol feeding on AD-relevant alterations in the liver of mice. **(A)** Expression of APP, LRP1, and other key proteins important in Aβ synthesis and transport in the liver following 4 weeks of intragastric alcohol feeding to mice. **(B)** Expression of LRP1 and APP mRNA following 4 weeks of intragastric alcohol feeding. LRP1 and LRP2-V2 represent two different primer variants that were examined by qPCR using SYBR Green. β-actin was used as control, as it appeared most stable compared to other control genes examined. Data represents the mean ± standard error, with *n* = 11–18 mice. Percent changes are calculated with 2^−ΔΔCT^. **(C)** Time course of LRP1 down regulation and APP up regulation in the liver following intragastric alcohol. Mice were fed alcohol intragastrically for 1, 2, and 4 weeks. All immunoblots shown are representative samples from four to six experiments. Densitometry for APP and LRP1 in the liver was performed using NIH Image J with β-actin being used as the loading control. Results are mean ± SD. *n* = 7–10 mice. ****p < 0.0001, ***p < 0.001, **p < 0.01, or *p < 0.05 vs. control.

In the liver, intragastric alcohol feeding significantly affected two important proteins associated with Aβ synthesis and transport: APP and LRP1 (Figure 3A). APP levels in the liver increased by ∼97% (*p* < 0.01) with intragastric alcohol feeding, while LRP1 levels in the liver declined by ∼46% (*p* < 0.01) as determined by immunoblotting. The levels of glycogen synthase kinase-3 (GSK) in the liver were not significantly altered with intragastric alcohol feeding. This increase in APP and decline in LRP1 in the liver with intragastric alcohol feeding was also observed by RT-qPCR (Figure 3B). The decline in LRP1 expression in ethanol fed mice was consistent across two selected primer sequences, with respective decreases of 27% (*p* < 0.05) and 34% (*p* < 0.05) compared to control animals. APP mRNA expression increased by ∼120% (*p* < 0.01) with alcohol feeding (Figure 3B), levels very similar to those observed with immunoblotting (Figure 3A). The changes in LRP1 and APP expression induced by alcohol were early events in intragastric alcohol feeding, as significant decline in LRP1 and APP was observed even at 2- and 4-weeks of intragastric alcohol feeding (Figure 3C).

### Mouse Aβ (1–42) levels in the brain, plasma, and the liver following intragastric alcohol feeding

**FIGURE 4:**
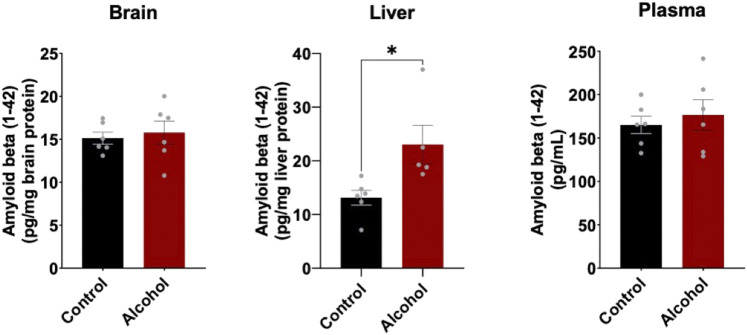
Mouse Aβ (1–42) levels in the brain, plasma, and liver following 4 weeks of intragastric alcohol feeding to mice. Levels of mouse Aβ (1–42) in brain and liver homogenate supernatants, as well as plasma samples were determined using ELISA. Results are mean ± SD. *n* = 6–7 mice. *p < 0.05 vs. control.

To determine the effect of alcohol-induced downregulation of hepatic LRP1 and upregulation of hepatic APP had on Aβ load in the body, Aβ (1–42) levels in brain, plasma, and liver were measured using ELISA (Figure 4). In the brain and plasma, no difference in Aβ (1–42) was observed after 4 weeks of intragastric alcohol feeding, likely due to the short nature of the alcohol feeding studies. In the liver, on the other hand, there was a significant increase in Aβ (1–42) (∼61%, *p* < 0.05) suggesting that increased alcohol-induced expression of APP, could be causing an increase in Aβ (1–42) production by the liver.

### Effect of different alcohol models on hepatic LRP1 and APP levels

**FIGURE 5:**
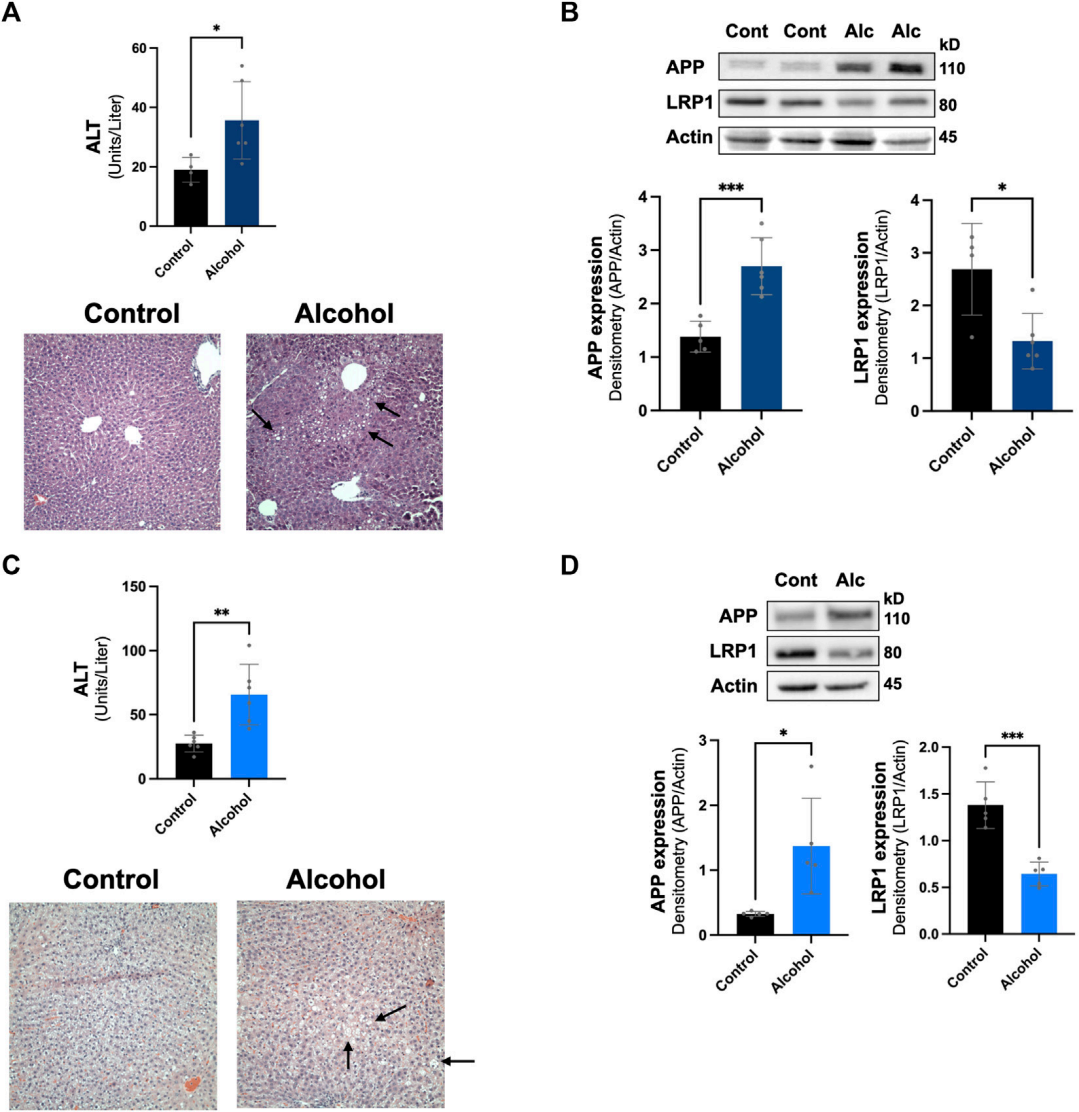
Effect of other alcohol feeding models on hepatic LRP1 and APP levels. The effect of the NIAAA binge model on liver injury and steatosis **(A)**, in addition to LRP1 and APP expression in the liver **(B)**. ALT, H&E histology (×200 magnification), and immunoblotting of mice liver occurred following NIAAA binge model (6 weeks oral alcohol feeding plus a binge). The arrows point to lipid droplets in the liver associated with steatosis. *n* = 4–6 mice. The effect of intragastric alcohol feeding to rats on liver injury and steatosis **(C)**, and hepatic LRP1 and APP expression in intragastric alcohol fed rats **(D)**. ALT, H&E histology, and immunoblotting of rat liver occurred following 6 weeks of intragastric alcohol feeding to rats (*n* = 5 rats). Densitometry for APP and LRP1 in the liver was performed using NIH Image J with β-actin being used as the loading control. Results are mean ± SD. ***p < 0.001, **p < 0.01, or *p < 0.05 vs. control.

The intragastric alcohol feeding model is the only method that allows for delivery of high doses of alcohol to animals that cause significant steatosis (fatty liver) and liver injury, seen in human alcoholic patients (Tsukamoto et al., 1985; Han et al., 2017). Oral alcohol feeding models (i.e., Lieber-DeCarli diet) which have been used in some rodent AD studies mainly produce some steatosis and cause low-to-moderate liver injury (Tsukamoto et al., 1985; Han et al., 2012; Han et al., 2017). The NIAAA binge alcohol model has become more widely utilized since it causes more liver injury and steatosis than oral alcohol feeding models, though still significantly less than the intragastric model (Bertola et al., 2013). We examined if alcohol feeding to mice using the NIAAA binge model also modulated LRP1 and APP in the liver. Figure 5A shows that the NIAAA binge model caused liver injury demonstrated by a significant elevation in ALT levels, although ALT levels were ∼5-fold lower than those observed with the intragastric alcohol feeding. Liver steatosis was also observed with the NIAAA binge model (Figure 5A), but generally at lower levels than observed with the intragastric alcohol feeding as previously discussed (Bertola et al., 2013). Although liver injury was lower with the NIAAA binge model, the decline in LRP1 (∼49%, *p* < 0.05) and increase in APP (∼110%, *p* < 0.01) was very similar to levels observed with the intragastric alcohol fed mice (Figure 5B).

We also examined whether alcohol-induced changes to hepatic LRP1 and APP levels were species specific by investigating changes in these proteins in rats that were fed alcohol intragastrically (Figures 5C,D). Rats are generally much more resistant to alcohol-induced liver injury than mice, and thus represents a different animal model of alcoholic liver disease (Shinohara et al., 2010). In agreement with previous studies, rats fed alcohol intragastrically had ∼3-fold lower ALT levels than mice fed alcohol intragastrically. Steatosis also appeared to be lower in rats than mice, which has been explored in other studies (Shinohara et al., 2010). Even though rats experienced less alcohol-induced liver injury then mice, hepatic LRP1 levels declined similarly to levels seen in mice (∼53%, *p* < 0.05), and APP expression increase was much more dramatic (∼320%, although with greater variability, *p* < 0.05). These results suggest that alcohol-induced decline in LRP1 and increase in APP was not species specific (occurring in both mice and rats) or alcohol feeding-model specific (occurring in both intragastric and oral NIAAA feeding models). Overall, alcohol-induced changes to hepatic LRP1 and APP occurred within a wide range of liver injury and steatosis.

### Modulation of hepatic LRP1 and APP in the ob/ob steatosis model

**FIGURE 6:**
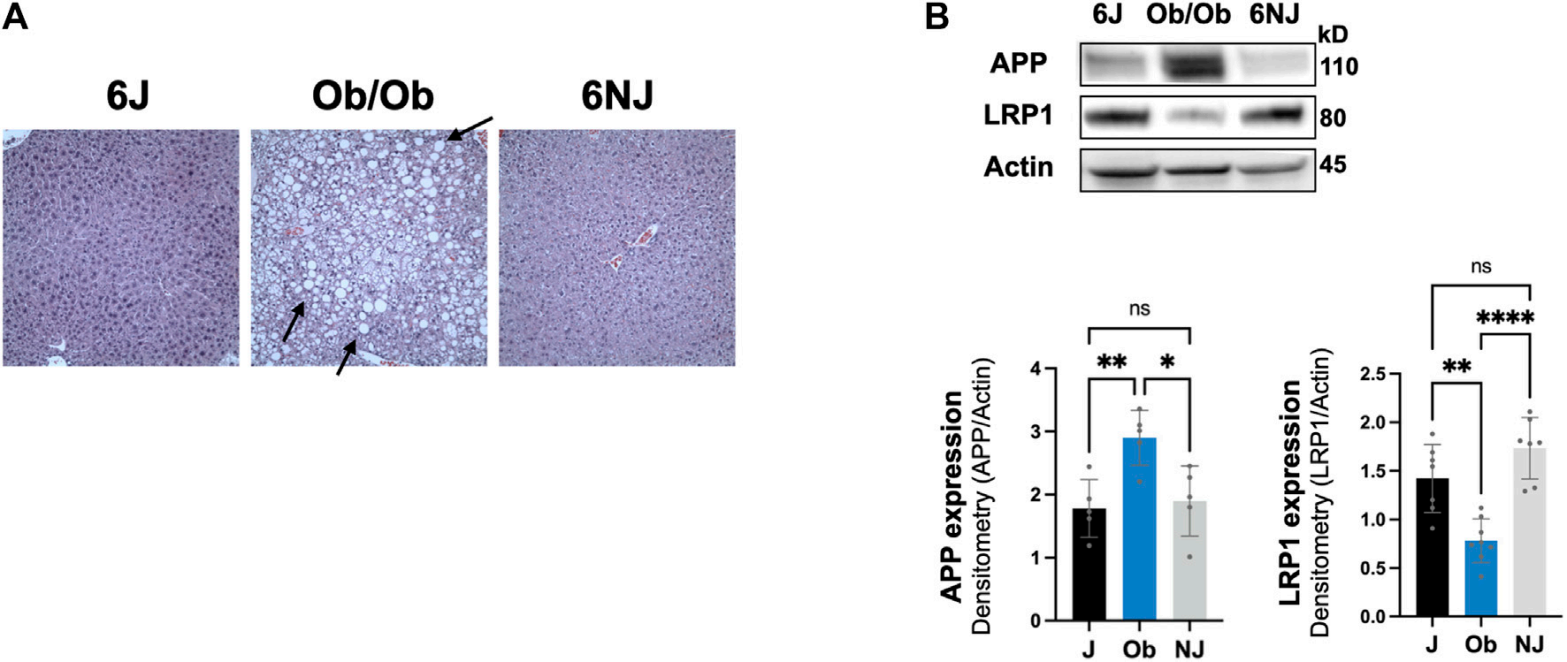
Expression of LRP1 and APP in ob/ob steatosis mice. **(A)** Representative H&E histology (×200 magnification) of ob/ob mice liver and control mice (J, NJ) at 20 weeks of age. The arrows point to lipid droplets in the liver associated with steatosis. **(B)** LRP1 and APP expression in the liver of ob/ob with advance steatosis. *n* = 6 mice per group. 6J and 6NJ represent two different control mice for ob/ob mice. Results are mean ± SD; ****p < 0.0001, **p < 0.01, or *p < 0.05 vs. J or NJ controls.

We next examined whether the downregulation of hepatic LRP1 and upregulation of hepatic APP was alcohol specific or due to liver pathology by investigating a non-alcohol model of steatosis, ob/ob mice. These mice have spontaneously developed a mutation in leptin, leading to increased food intake, obesity, and steatosis (Sharma et al., 2010; Rull et al., 2014). Ob/ob mice have even more steatosis than intragastric alcohol fed mice (Figure 6A), which has been well documented (Rull et al., 2014; Garcia et al., 2018). Ob/ob mice had a comparable significant decline in LRP1 (∼45% vs. 6J, ∼60% vs. NJ, *p* < 0.05) similar to the levels observed in the alcohol models and had a significant increase in APP (∼59% vs. 6J, ∼51% vs. NJ, *p* < 0.05), which was somewhat lower than that observed with alcohol feeding models (Figure 6B). These results suggest that changes in hepatic LRP1 and APP are not solely due to alcohol, and that steatosis may play a significant role in the regulation of these two proteins in the liver.

## Discussion

The consumption of chronic alcohol is a known risk factor for various forms of dementia and the development of AD (Fratiglioni et al., 1993; Heymann et al., 2016). The main hallmarks of AD are Aβ deposition, phosphorylated tau containing neurofibrillary tangles, neuronal loss and microgliosis (Crews and Masliah, 2010). Accordingly, utilizing the intragastric alcohol feeding model, the model which can deliver highest amount of alcohol to mice and rats, we studied the effect of alcohol consumption on proteins involved in Aβ synthesis and transport, neuronal health, and microgliosis. We observed that high levels of alcohol feeding resulted in a significant decline in the number of microglial cells in the cortex. This finding is consistent with an increase in dystrophic microglia and a reduction in the number of microglia with alcohol consumption in rats (Marshall et al., 2020). The implications of these findings for AD can be significant given the complex and contrasting roles played by microglia (Leng and Edison, 2021). In the AD brain, microglia are found surrounding Aβ plaques and microglial recognition of Aβ can trigger microglial activation that can result in Aβ phagocytosis and clearance (Sarlus and Heneka, 2017; Leng and Edison, 2021). Additionally, these plaque-surrounding microglia were recently shown to form a physical barrier around the Aβ plaques protecting against neuronal damage (Spangenberg et al., 2019). Therefore, a decrease in microglia with alcohol feeding could impact Aβ and neuronal homeostasis in the brain (Sarlus and Heneka, 2017; Hansen et al., 2018), and this idea is further supported by a reduction in Aβ phagocytosis by alcohol-treated microglia (Kalinin et al., 2018). In contrast, Aβ mediated microglial activation can also trigger cytokine release and neuroinflammation, which can in turn increase Aβ production through the upregulation of β-secretase (Sarlus and Heneka, 2017; Leng and Edison, 2021). A decrease in microglia with alcohol feeding can therefore also potentially reduce neuroinflammation and Aβ production. Further studies in transgenic mouse models of AD are needed to gauge the exact impact of alcohol-induced decline of microglial number on AD pathology. How alcohol reduces microglial cell number in the brain remains unknown, but it has been suggested that alcohol may be directly cytotoxic to microglia (Marshall et al., 2020). Interestingly, we observed a significant inverse relationship between liver injury (ALT levels) and microglial levels in the cortex; with greater liver injury, there was a greater decline in microglial cell number. This suggests a potential liver-brain axis regulating microglial cells in the brain. Liver injury may trigger the release of various toxins (e.g., reactive oxygen species, acetaldehyde), cytokines that promote neuroinflammation, or other signaling molecules that decrease microglial replication or promote microglial apoptosis (Han et al., 2006; Venkataraman et al., 2017). However, the possibility that liver injury and the decline in microglial cells occurred concurrently due to poorer capacity to metabolize alcohol or other factors cannot be ruled out. With respect to endogenous APP and endogenous mouse Aβ (1–42) expression, we did not observe an increased expression of APP or Aβ in the brain, which had been reported in previous oral or binge alcohol feeding studies (Kim et al., 2011; Huang et al., 2018). Our work examined APP and Aβ in total brain of wild-type mice, while other studies used transgenic AD mice (APP23/PS45 mice) where human APP and PSE1 are overexpressed (Huang et al., 2018) or studied APP increase in specific regions of the brain (cerebellum, hippocampus, and striatum), and not total brain homogenates. It is quite possible that APP and/or Aβ upregulation is brain-region specific following intragastric alcohol feeding, and future work will be needed to determine brain-region specific APP and Aβ expression following intragastric alcohol feeding.

**FIGURE 7:**
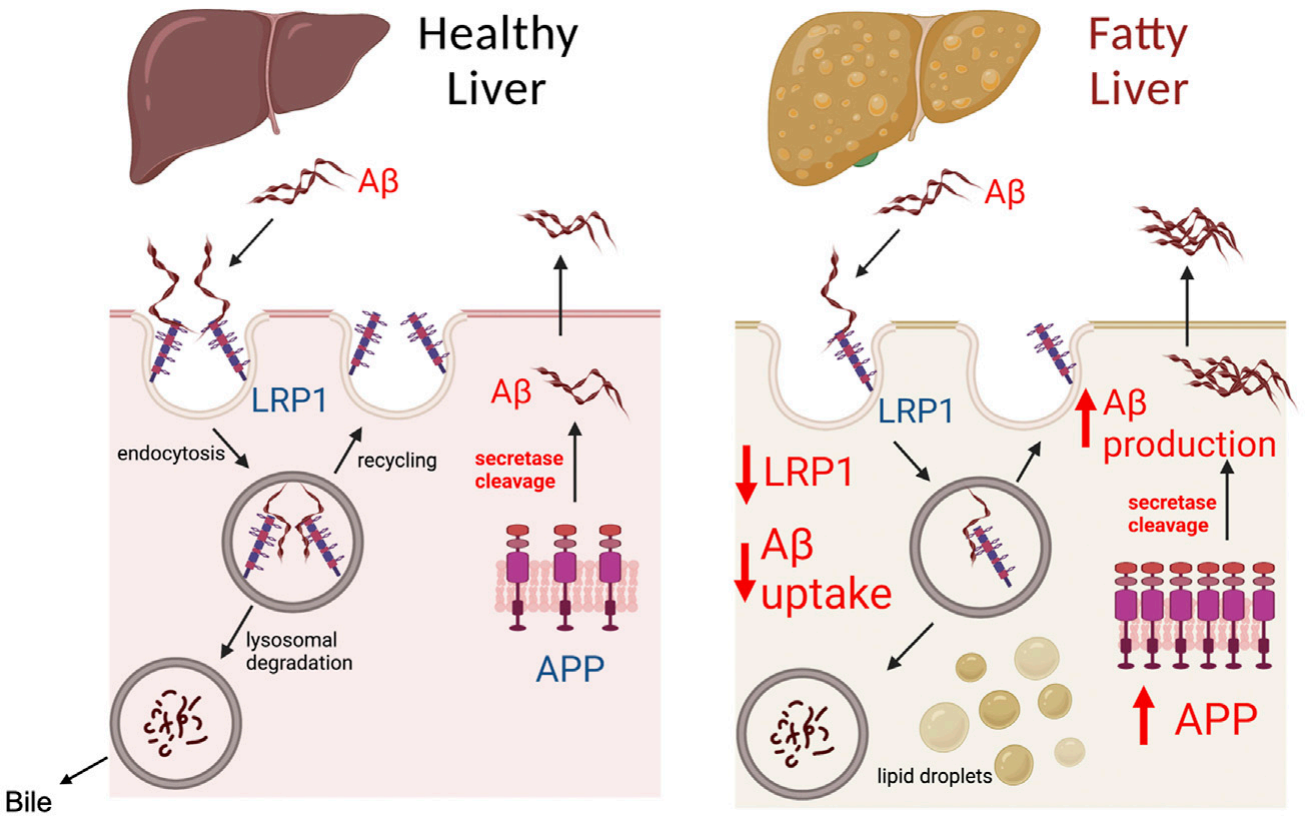
Model of Aβ processing by healthy and fatty liver. The liver plays a significant role in determining Aβ load in the peripheral organs and blood, as both a source of Aβ and by clearing Aβ. LRP1 in the liver has been identified as the major receptor responsible for Aβ clearance from the blood. Aβ binding to LRP1 is believed to trigger endocytosis and uptake of Aβ inside hepatocytes, where it is degraded by lysosomes, and secreted in bile. Modulation of LRP1 in the liver has been shown to affect the capacity of the liver to clear Aβ from the periphery. The liver also contains APP, which through the action of secretases such as PSE2, can be cleaved to Aβ causing its release into circulation. A healthy liver is likely to consume or produce low levels of Aβ, unless there are genetic mutations such as PSE2 mutations. In the case of fatty liver (steatosis), our work demonstrates that LRP1 levels are inhibited by ∼50% and APP expression is increased 2-fold. The decreased LRP1 and increased APP expression during liver steatosis likely makes the liver an important source of Aβ in the blood and liver, which can increase Aβ levels in the brain to promote AD. Created with BioRender.com.

The most significant finding of our work was that chronic alcohol feeding or obesity causes a major decline in hepatic LRP1 levels while substantially increasing hepatic APP levels. These two changes are likely to have a significant effect on hepatic processing of Aβ, possibly altering the liver from being a remover or minor source of Aβ to an important source of Aβ in the periphery (Figure 7). The notion that the liver becomes an important source of Aβ following steatosis is supported by the following observations. First, liver specific overexpression of human APP has been shown to increase Aβ levels in the periphery and the brain to promote AD pathology in mice (Lam et al., 2021). The human Aβ produced in the liver of these transgenic mice was not observed in the brain of mice at 6 months of age, but became significant at 1 year, suggesting it takes a significant amount of time for Aβ produced in the liver to accumulate in the brain. A 2-fold increase in hepatic APP expression observed in our studies following alcohol feeding is similarly likely to increase Aβ production by the liver, but likely would take time to make a significant impact in the periphery and brain. Consequently, we only observed increases in Aβ levels in the liver, and not in the plasma or brain following alcohol feeding, probably due to the short duration of our studies. Unfortunately, it is very difficult to perform lengthy alcohol feeding studies in animals, but further studies examining steatosis caused by high fat feeding on hepatic Aβ production are being performed.

In addition to upregulation of APP, the downregulation of LRP1 associated with steatosis is likely to significantly impact the ability of the liver to clear Aβ from the periphery (Tamaki et al., 2006; Zlokovic et al., 2010). Previous studies have demonstrated a link between hepatic function and peripheral Aβ clearance, with liver injury being associated with elevated peripheral Aβ in patients (Wang et al., 2017). The mechanism of how liver injury could affect Aβ clearance has not been identified. Our data suggests that a 50% decline in hepatic LRP1 expression associated with alcohol feeding and in ob/ob mice may explain the previously observed relationship between hepatic function and Aβ clearance. LRP1 has been demonstrated to be the major receptor in the liver involved in the clearance of Aβ from the periphery (Tamaki et al., 2006; Zlokovic et al., 2010; Mohamed and Kaddoumi, 2013). Suppression of hepatic LRP1 has been shown to decrease Aβ uptake by the liver (Tamaki et al., 2006), while its upregulation by the root extract *Withania somnifera* (WS) was shown to decrease plasma- and brain-Aβ levels and improve cognitive function in aged AD mice (Sehgal et al., 2012). LRP1 expression in the liver has been shown to significantly decline with age, which also corresponded with decline in Aβ clearance by the liver (Tamaki et al., 2006).

The fact that both alcohol feeding models (intragastric to mice and rats, NIAAA binge) and obesity caused the downregulation of LRP1 and upregulation of APP suggests that steatosis may be the uniting factor in the dysregulation of these two genes in the liver. In support of this notion, gene profiling of non-alcoholic fatty liver disease (NAFLD) patients showed that APP gene expression in the liver was increased (Qi et al., 2017). In another study, high fat diet feeding to wild-type and AD transgenic mice (APP-Tg) was found to decrease LRP1 levels in the brain, however, the LRP1 levels in the liver were not examined (Kim et al., 2016). However, ob/ob mice and NAFLD are associated with some increases in ALT and liver injury, so the distinction between liver injury and steatosis in the regulation of hepatic LRP1 and APP may be difficult to untangle. Liver steatosis is a disease associated with aberrant lipid metabolism involving many signaling pathways including transcription factors such as p63 and stress signaling including activation of JNK (Win et al., 2021; Da Silva Lima et al., 2022). The lipid droplets and fatty acids associated with liver steatosis in turn can affect many signaling pathways including insulin signaling to promote diabetes mellitus type II (Shannon et al., 2021). Thus, signaling pathways involved in triggering steatosis and signaling pathways altered by lipid accumulation during steatosis could be involved in regulation of LRP1 and APP. The alcohol-induced signaling pathways that modulate hepatic APP and LRP1 occurred within 1 week of intragastric alcohol feeding, suggesting it is an early event with alcohol feeding. More detailed time course experiments are needed to determine when hepatic APP and LRP1 signaling pathways are modulated by alcohol in the various models.

LRP1 has been shown to be regulated by various signaling pathways including SREBP2, Sp1/3, and PPARγ (Gauthier et al., 2003; Costales et al., 2010; Aledo et al., 2014). SREBP2 may play a key role in the downregulation of hepatic LRP1 during alcohol feeding. Chronic alcohol feeding has been shown to activate SREBP2 (cleavage and translocation to the nucleus) in the liver (Ji et al., 2006), and SREBP2 has been shown to bind to the SRE sequence located in the 5′-UTR of LRP1 and downregulate its expression (Llorente-Cortes et al., 2007; Costales et al., 2010). qPCR data shows only a 27%–34% decline in LRP1 mRNA expression, suggesting the ∼50% decline in LRP1 protein expression may also be due to translational or post-translational changes. LRP1 in the liver has been shown to be cleaved by proteases such as BACE (Von Arnim et al., 2005). It is possible that soluble LRP1 (sLRP1) in plasma, a peripheral “Aβ-sink,” may originate from the liver following cleavage by proteases (Sagare et al., 2007). The post-translational modification of LRP1 in liver may contribute to the reduction of LRP1 that occurs with steatosis, which requires further investigation. The promoter for APP has binding sites for a number of transcription factors, including a heat shock control element, SP-1, AP-1, and AP-4 sites (Salbaum et al., 1988; Quitschke and Goldgaber, 1992). In HeLa cells, alcohol treatment and other stressors (e.g., temperature, sodium arsenite) have been shown to upregulate APP expression through activation of heat shock elements (Dewji et al., 1995; Dewji and Do, 1996). A similar mechanism of heat shock factors enhancing APP transcription in the liver following alcohol treatment may also be occurring. Other transcriptional factors such as AP-1 may also play a role in upregulation of APP observed in our studies, as AP-1 activation has been shown to play an important role in liver steatosis (Hasenfuss et al., 2014). Clearly, further studies will be needed to characterize the signaling pathways that modulate hepatic LRP-1 and APP in alcohol-induced and obesity-induced liver steatosis.

## Conclusion

Alcohol and obesity are two conditions known to cause steatosis in the liver and have also been linked to AD. Heavy alcohol consumption is a known risk factor for various forms of dementia and the development of AD (Fratiglioni et al., 1993; Heymann et al., 2016). Similarly, obesity, particularly in midlife, is a well-characterized risk factor for the development of AD (Whitmer et al., 2008; Gustafson et al., 2012). Previous studies attempting to characterize the link between alcohol and obesity have primarily focused on the brain. Our work shows that the liver may also play a very important role in the pathology of AD caused by obesity and chronic alcohol intake. We found that liver injury (ALT levels) was inversely correlated with microglia numbers suggesting a possible liver-brain crosstalk. We also demonstrate that liver steatosis triggers a decline in LRP1 expression, while simultaneously increasing APP expression in the liver. These changes likely decrease the liver’s capacity to take up Aβ from the periphery and enhance its potential to produce Aβ and promote AD.

## Data Availability

The original contributions presented in the study are included in the article/Supplementary Material, further inquiries can be directed to the corresponding author.

## Abbreviations

Aβ, amyloid-beta; AD, Alzheimer’s disease; ALT, alanine transaminase; APP, amyloid precursor protein; LRP1, lipoprotein receptor-related protein 1; NAFLD, non-alcoholic fatty liver disease; PSE1, presenilin 1; PSE2, presenilin 2; SYP, synaptophysin
