# Supplementary material for: Modulation of hepatic amyloid precursor protein and lipoprotein receptor-related protein 1 by chronic alcohol intake: Potential link between liver steatosis and amyloid-β
Source: Front Physiol. 2022 Sep 15;13:930402. doi: 10.3389/fphys.2022.930402 (PMC9520570; doi:10.3389/fphys.2022.930402)
Supplement: Supplementary file 1 [file DataSheet1.PDF]

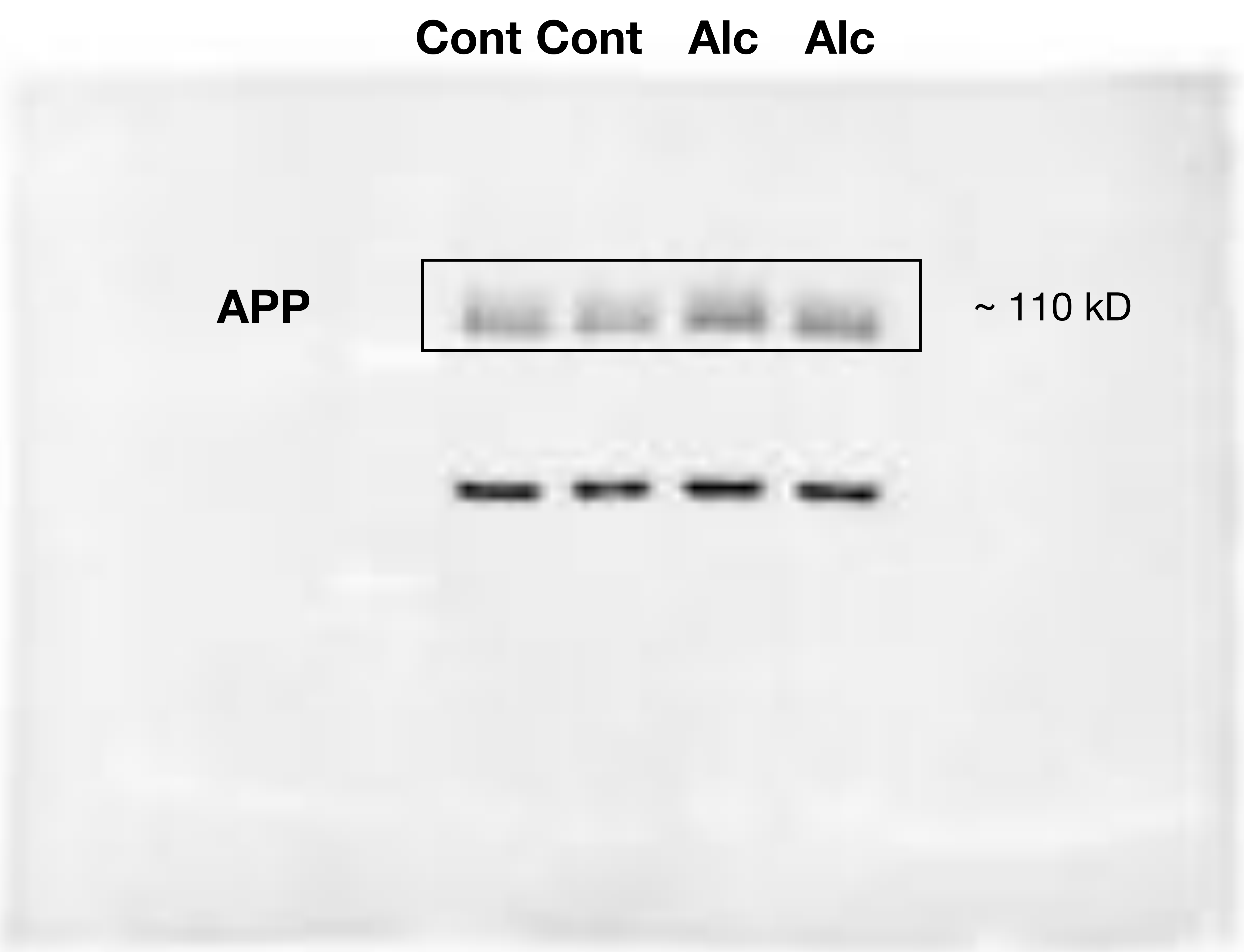

Full gel from Figure 1A

Supplementary Figure 1A

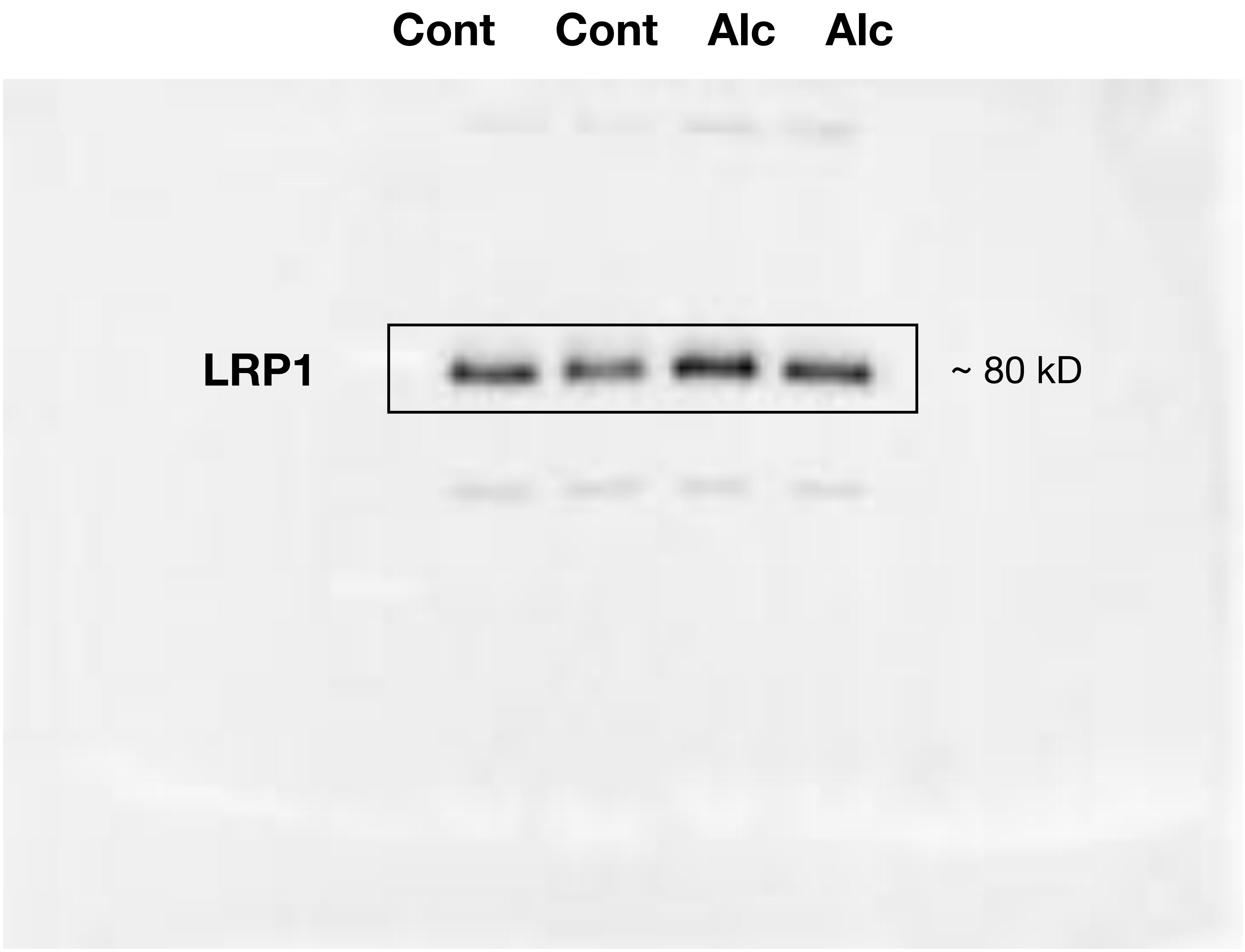

Full gel from Figure 1A

Supplementary Figure 1A

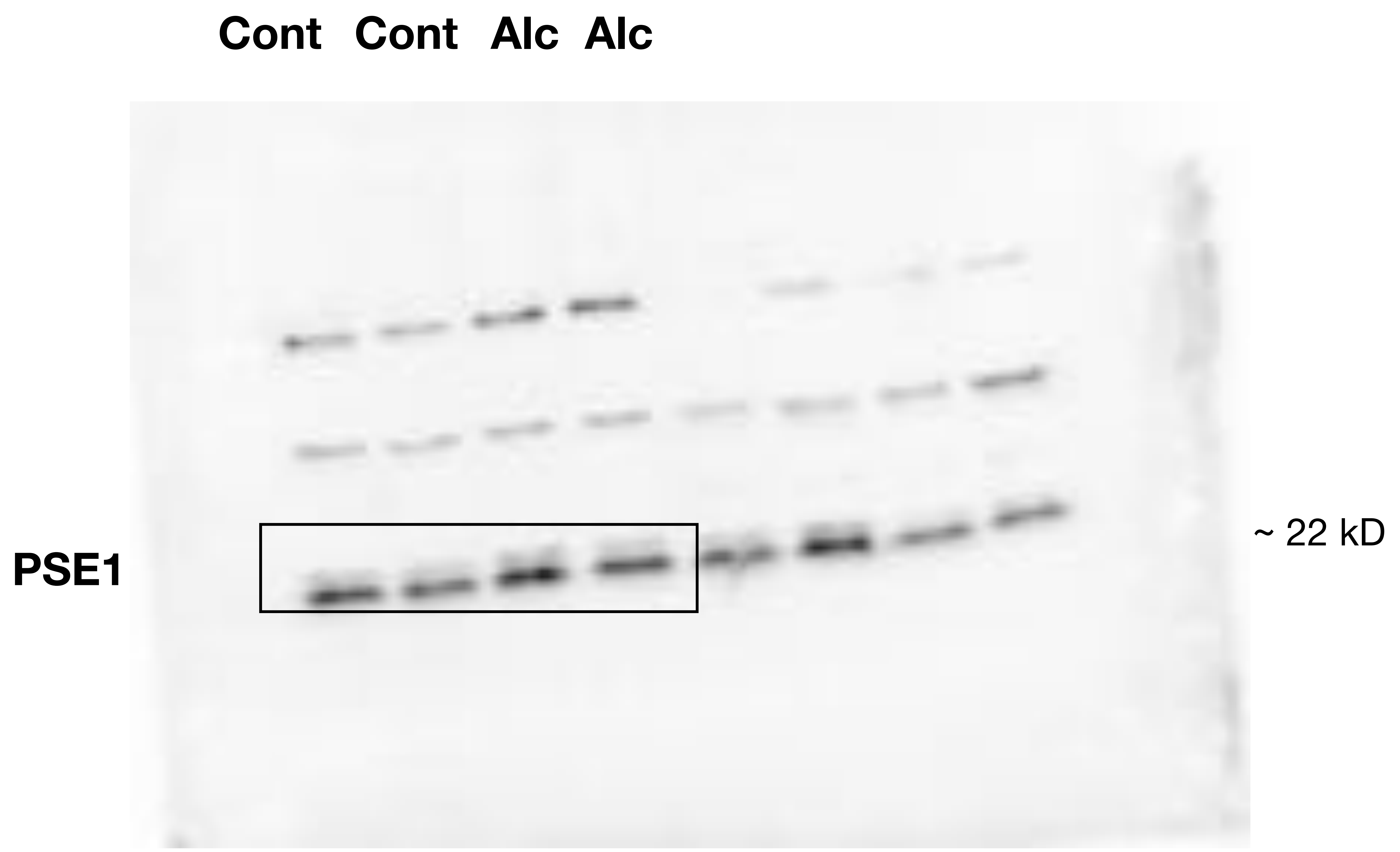

PSE1 and PSE2 have same MW so different gel was used for PSE1 to confirm its identify

Full gel from Figure 1A

Supplementary Figure 1A

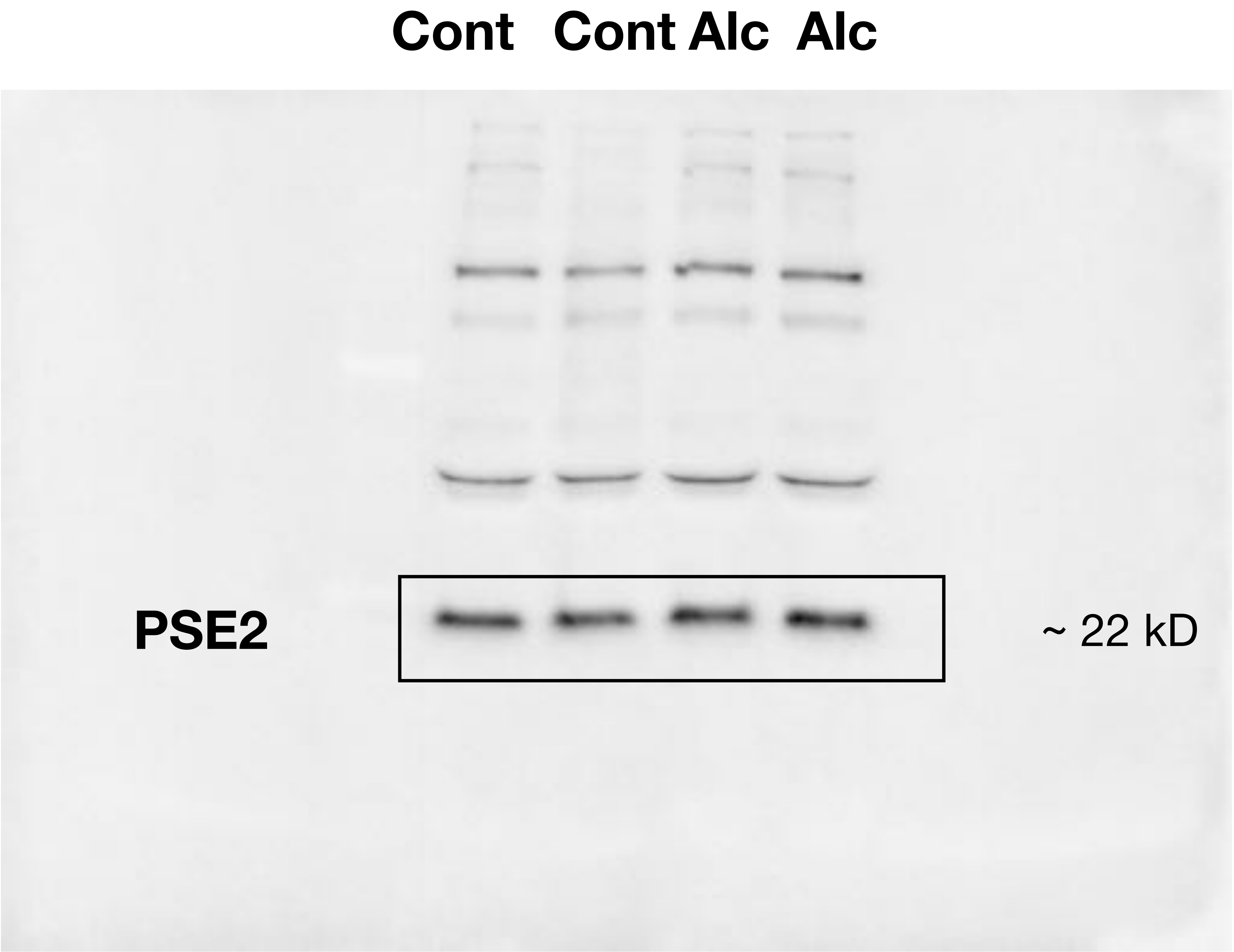

Full gel from Figure 1A

Supplementary Figure 1A

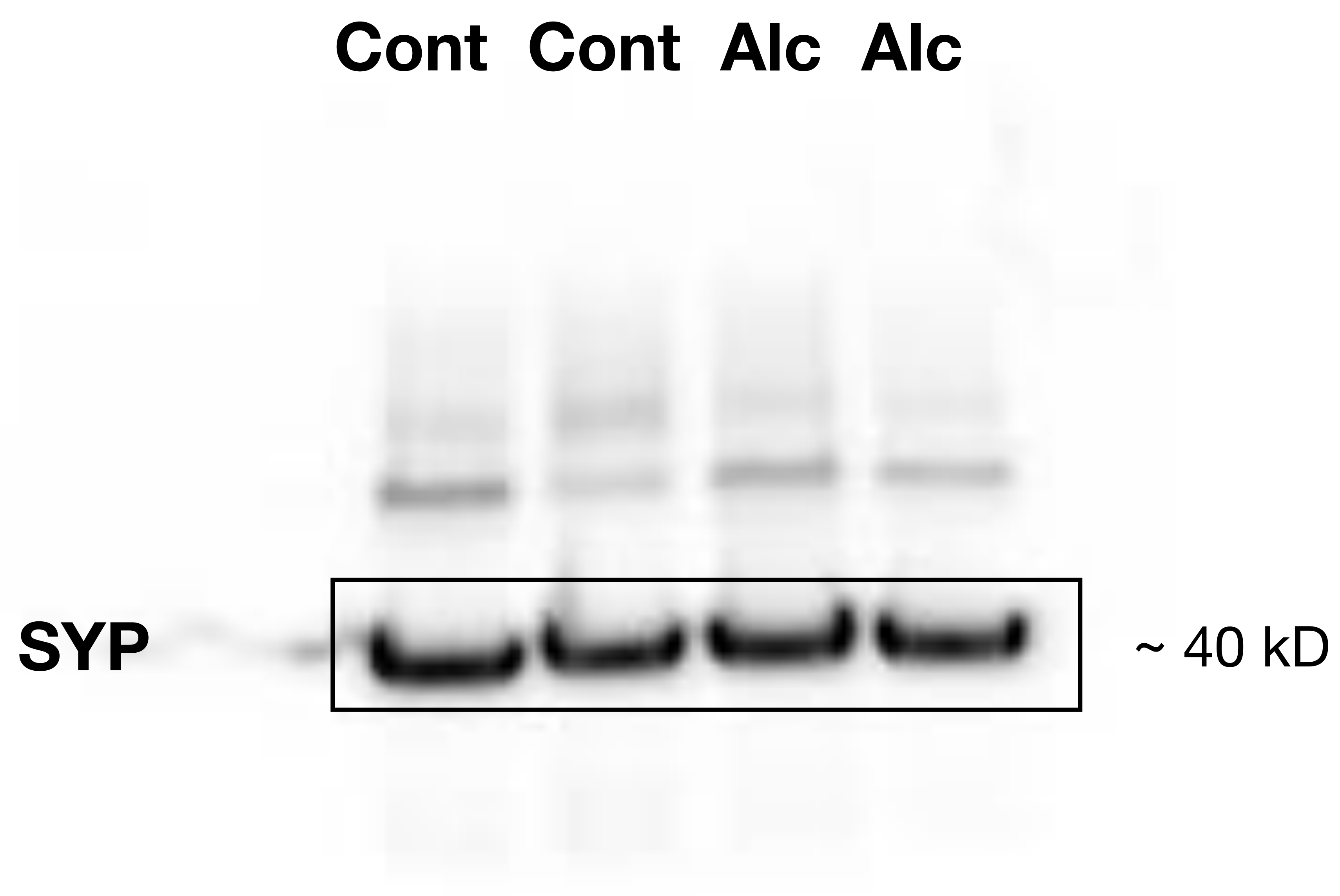

Full gel from Figure 1A

Supplementary Figure 1A

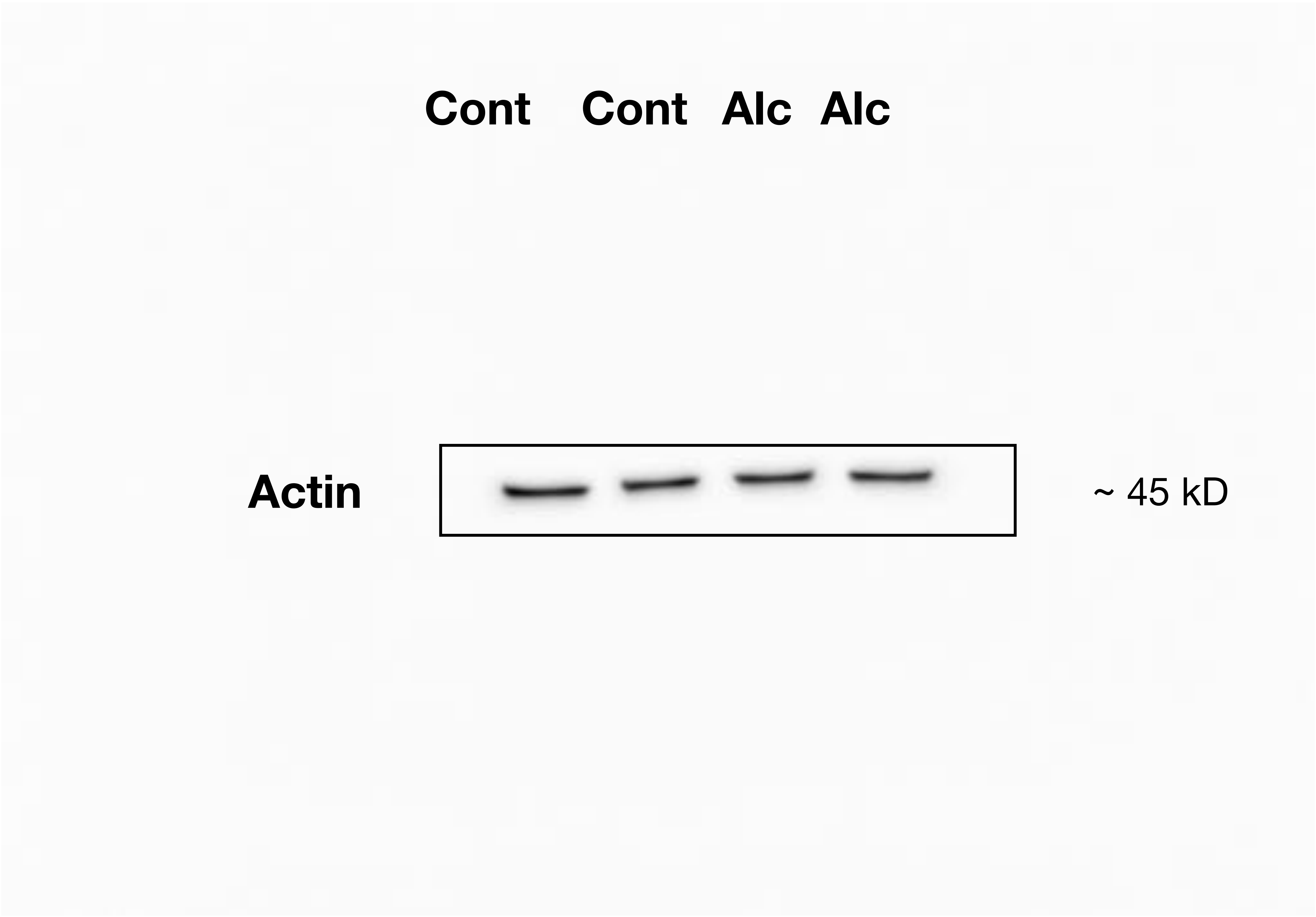

Full gel from Figure 1A

Supplementary Figure 1A

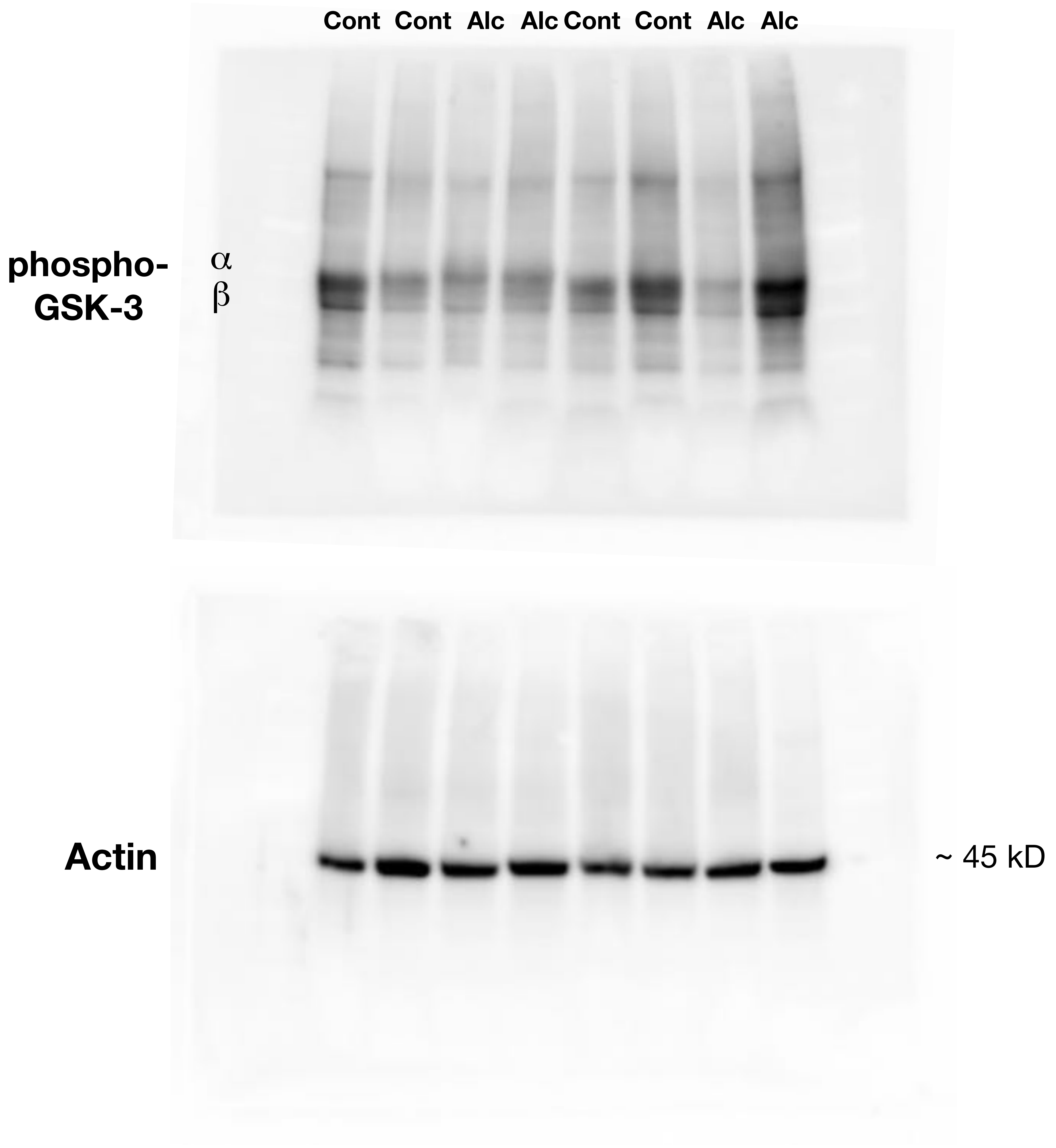

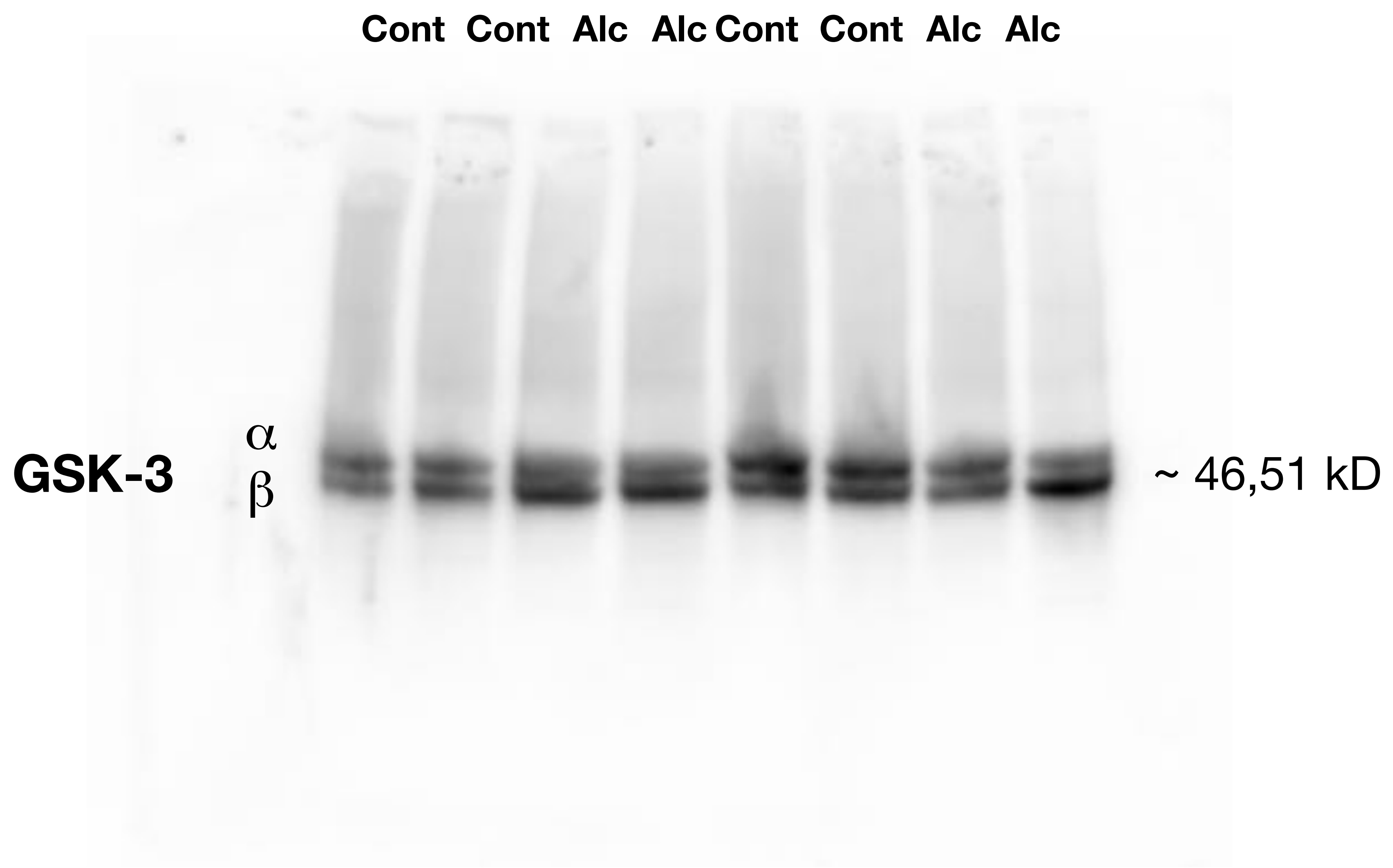

Immunoblots for phospho-GSK and GSK in the liver following intragastric alcohol feeding. Protein electrophoresis occurred on 8-12% SDS polyacrylamide gels, proteins were transferred to nitrocellulose or PVDF membranes, and blots were blocked with 5% (w/v) nonfat milk dissolved in Tris-buffered saline (TBS) with Tween-20. Antibodies to phospho-GSK (cat #9331), GSK (cat #5676) and actin (cat #3700) were obtained from Cell Signaling Technology.

Supplementary Figure 3A

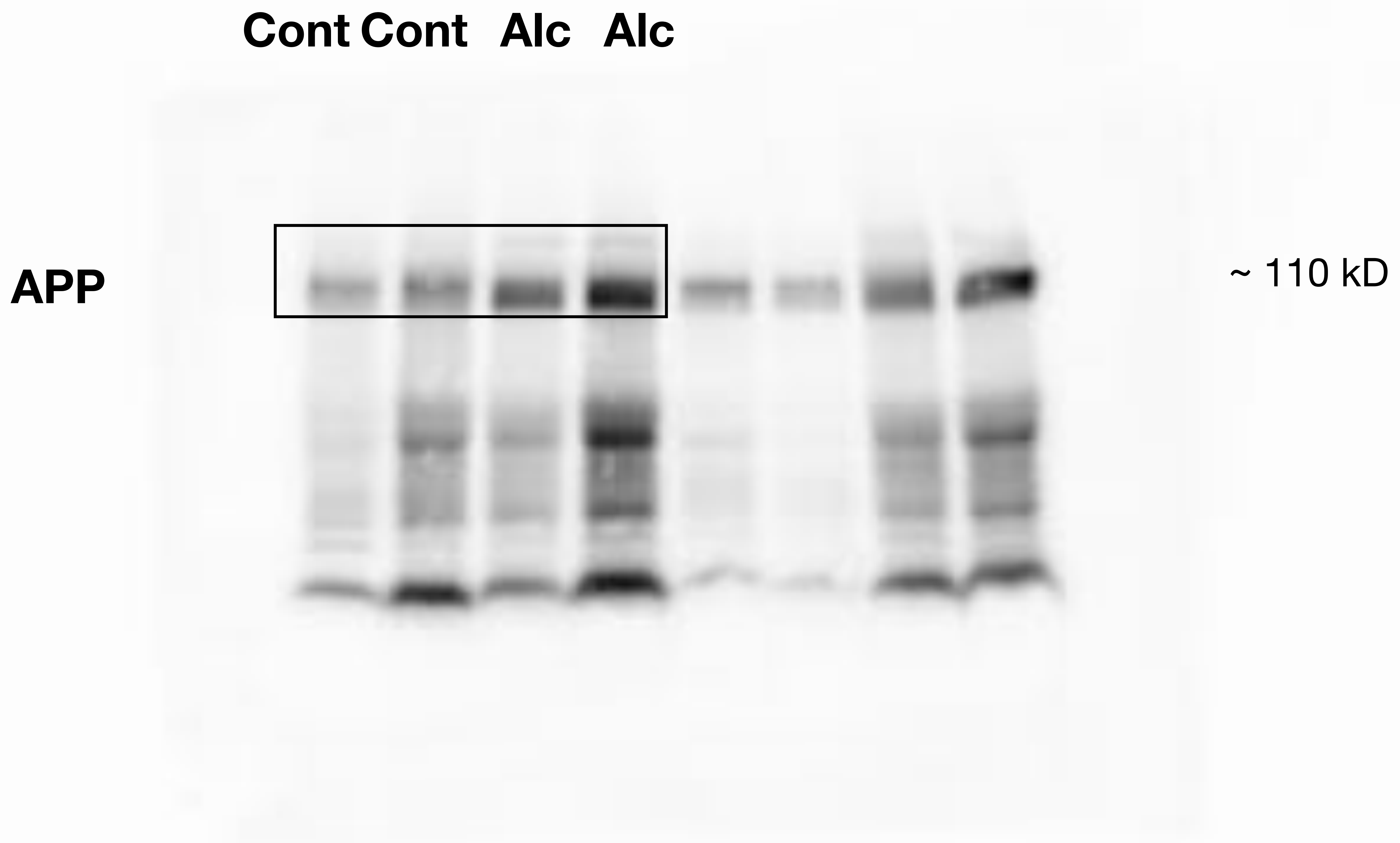

Full gel from Figure 3A

Supplementary Figure 3A

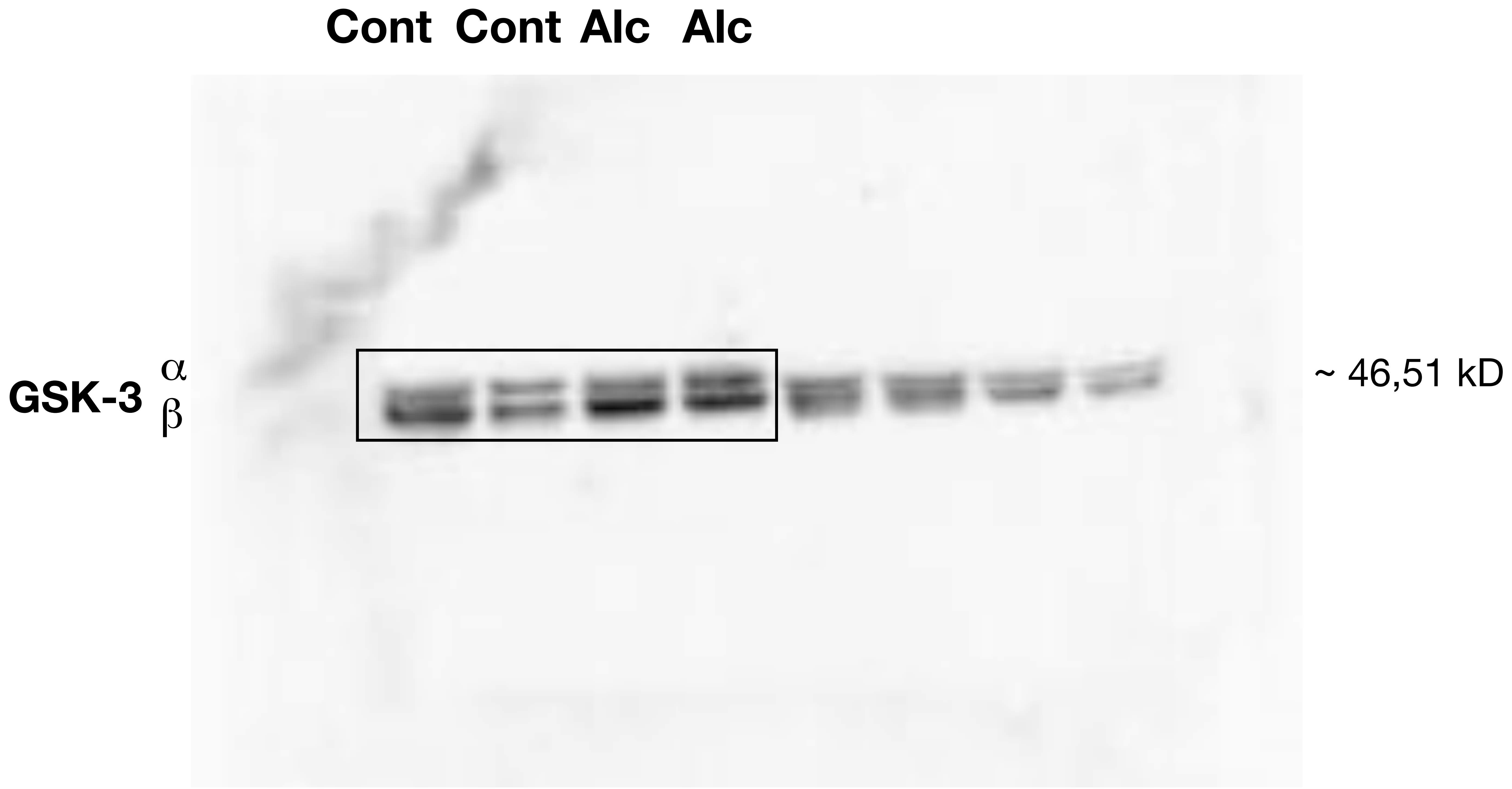

Full gel from Figure 3A

Supplementary Figure 3A

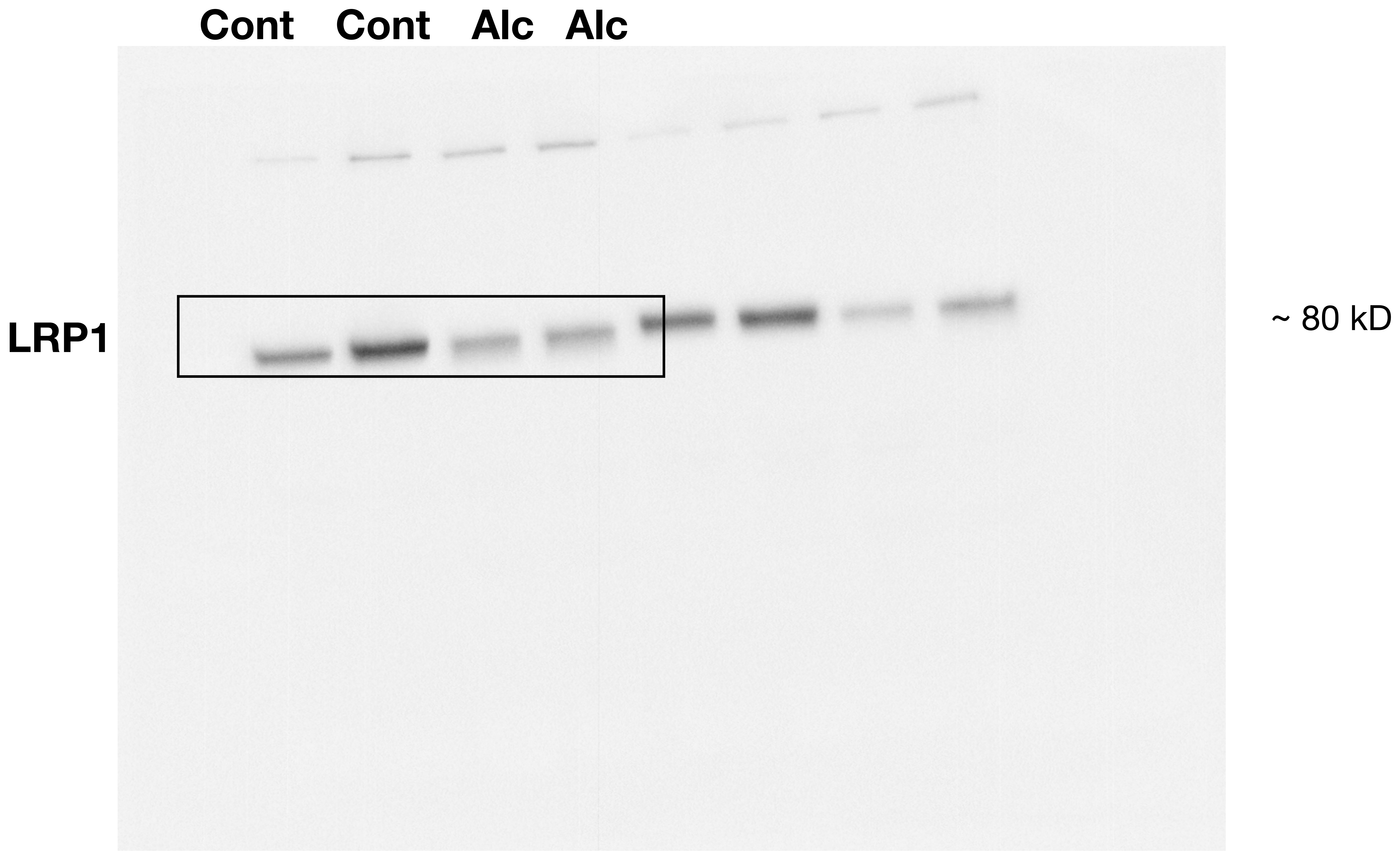

Full gel from Figure 3A

Supplementary Figure 3A

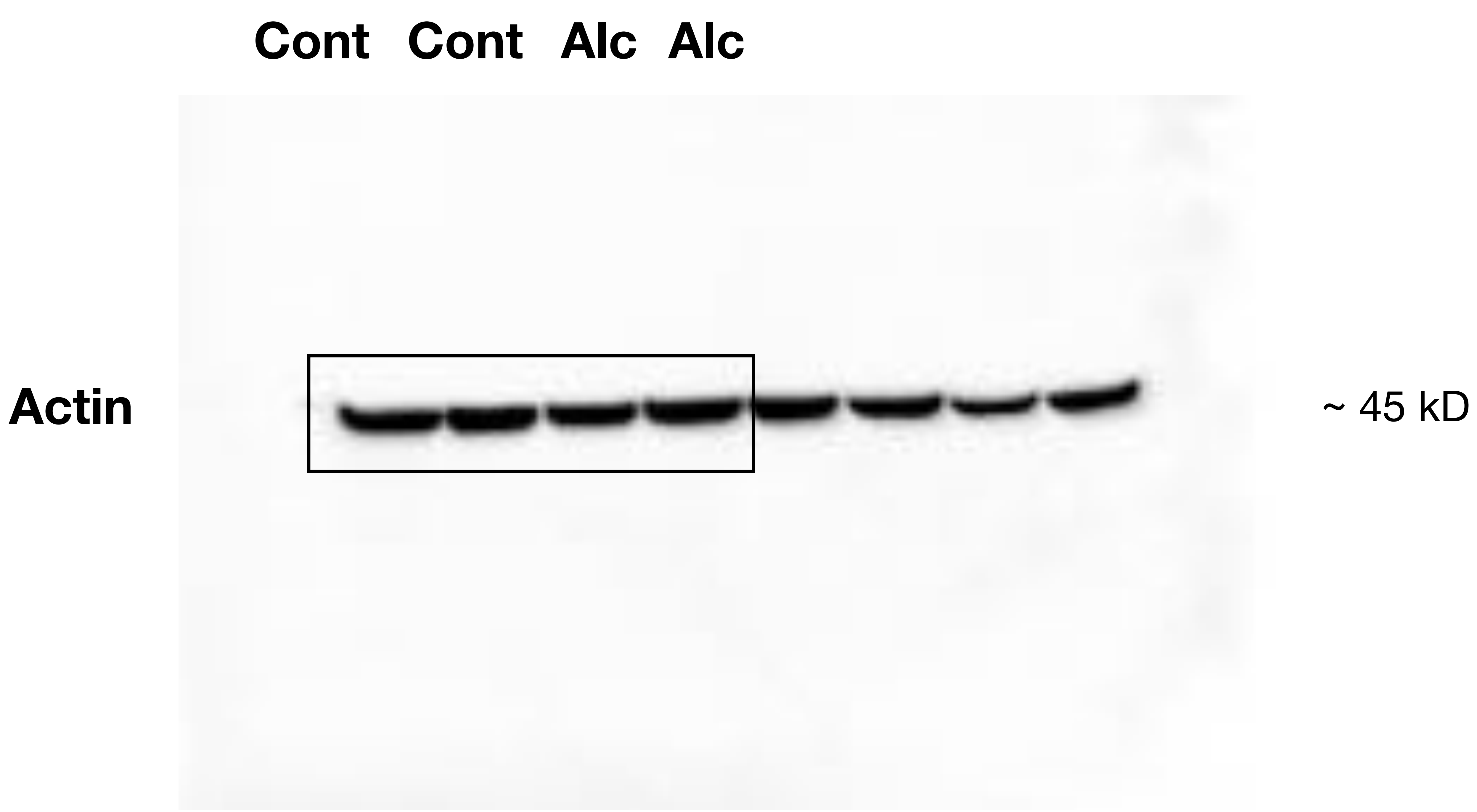

Full gel from Figure 3A

Supplementary Figure 3A

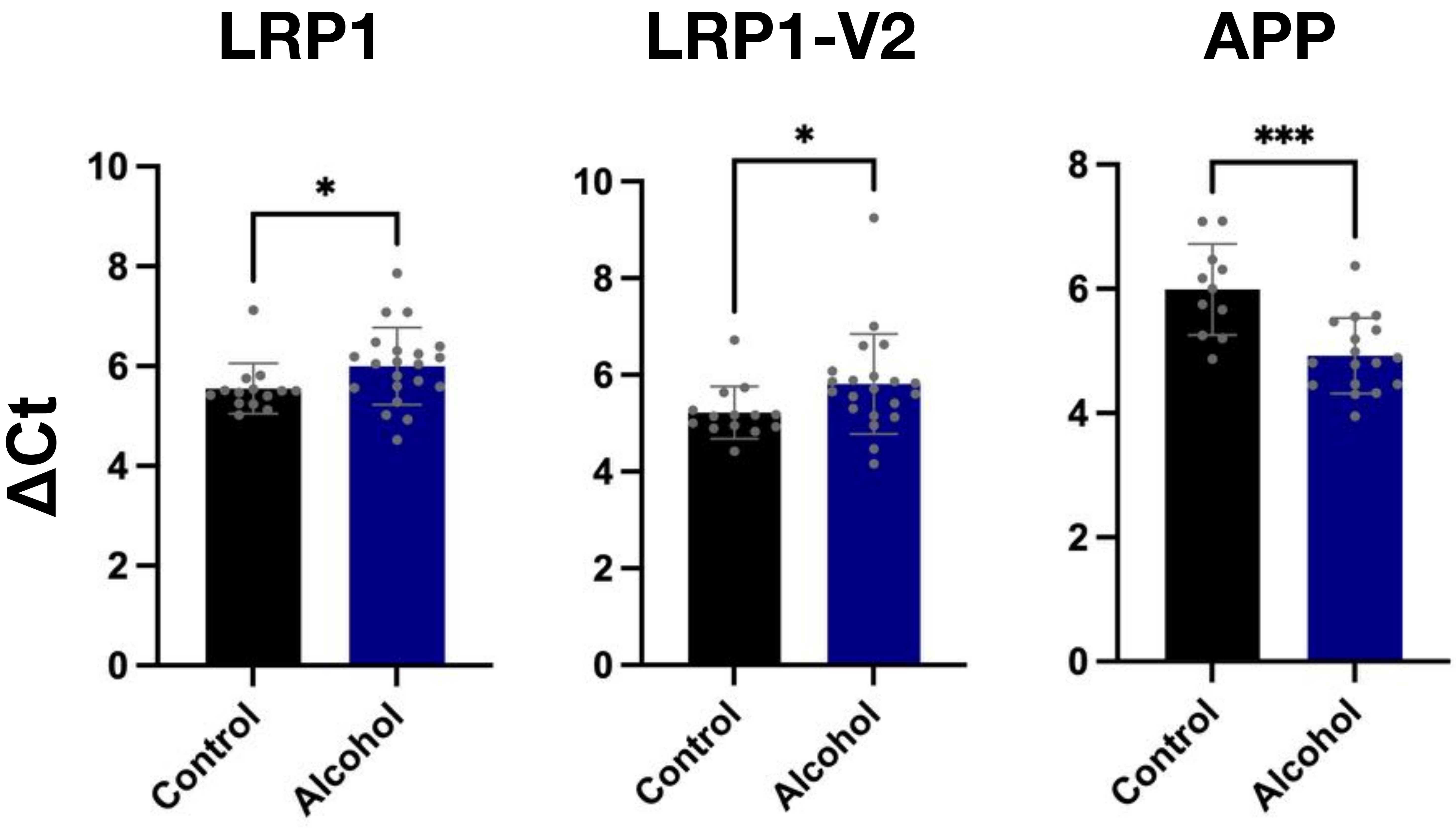

Effect of alcohol on hepatic LRP1 and APP expression assessed by RT-PCR. The ΔCt value ($Ct_{target\ gene}$ - Ct_{actin}) of LRP1 and APP following RT-qPCR are shown. The ΔCt values demonstrate that LRP1 amplification in alcohol fed mice was higher than control mice, suggesting alcohol feeding decreased LRP1 mRNA expression. Conversely APP amplification was significantly lower with alcohol feeding, suggesting increased APP mRNA expression in the liver following alcohol feeding. LRP1 and LRP2-V2 represent two different primer variants that were examined by qPCR using SYBR Green. Results are mean ± SD. N = 11-18 mice.* P < 0.05 or *** P < 0.001 versus control.

Supplementary Figure 3B

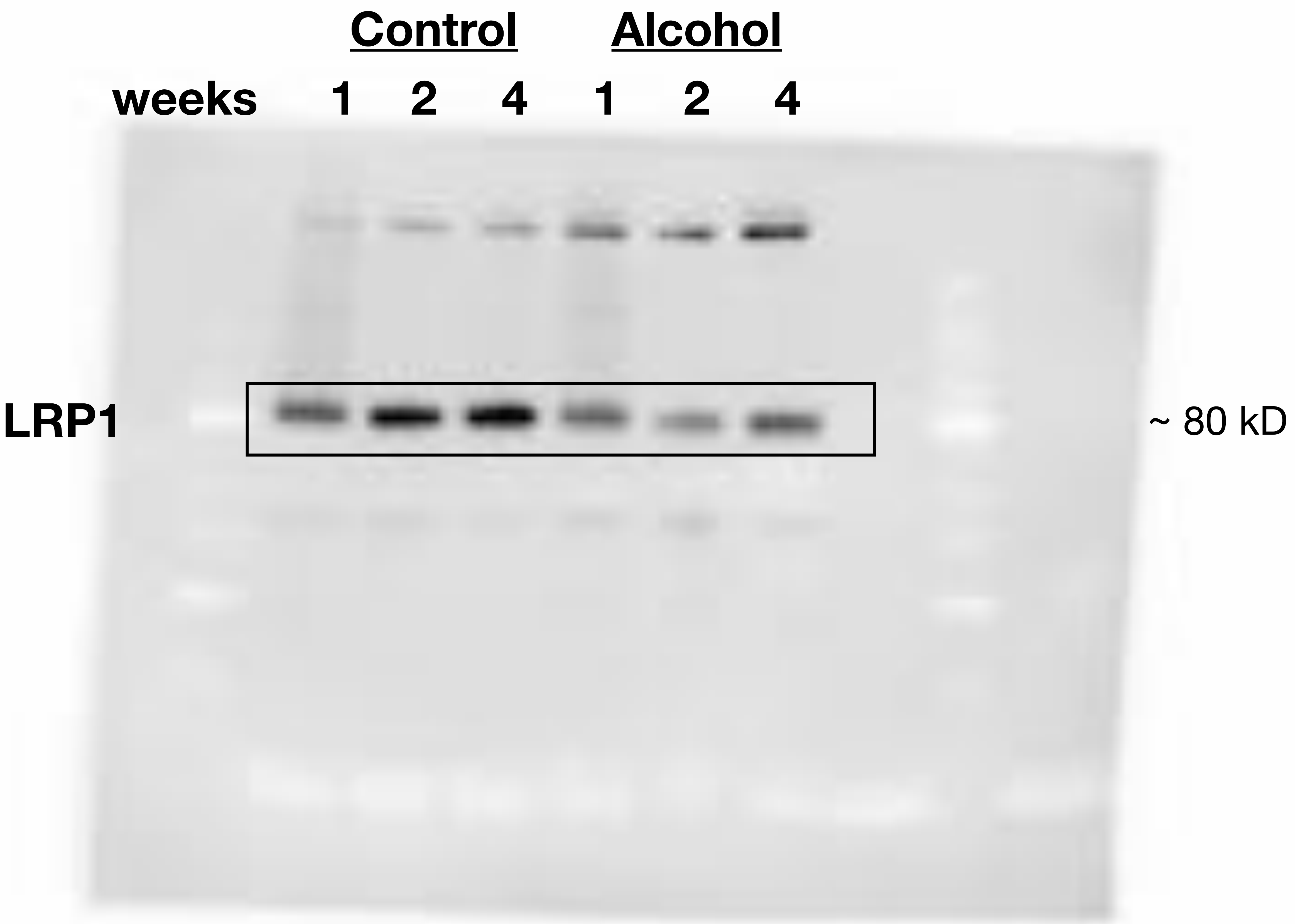

Full gel from Figure 3C

Supplementary Figure 3C

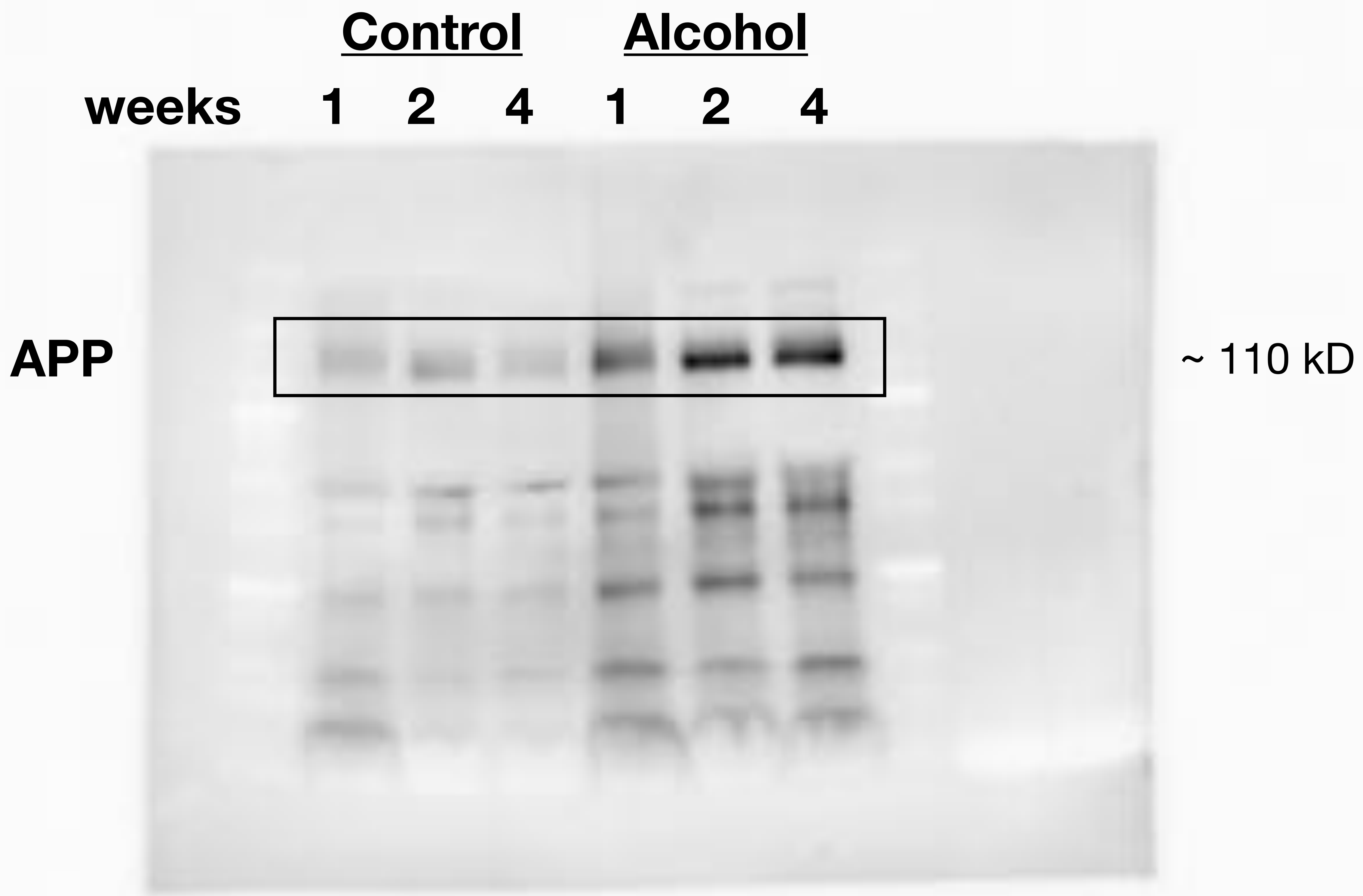

Full gel from Figure 3C

Supplementary Figure 3C

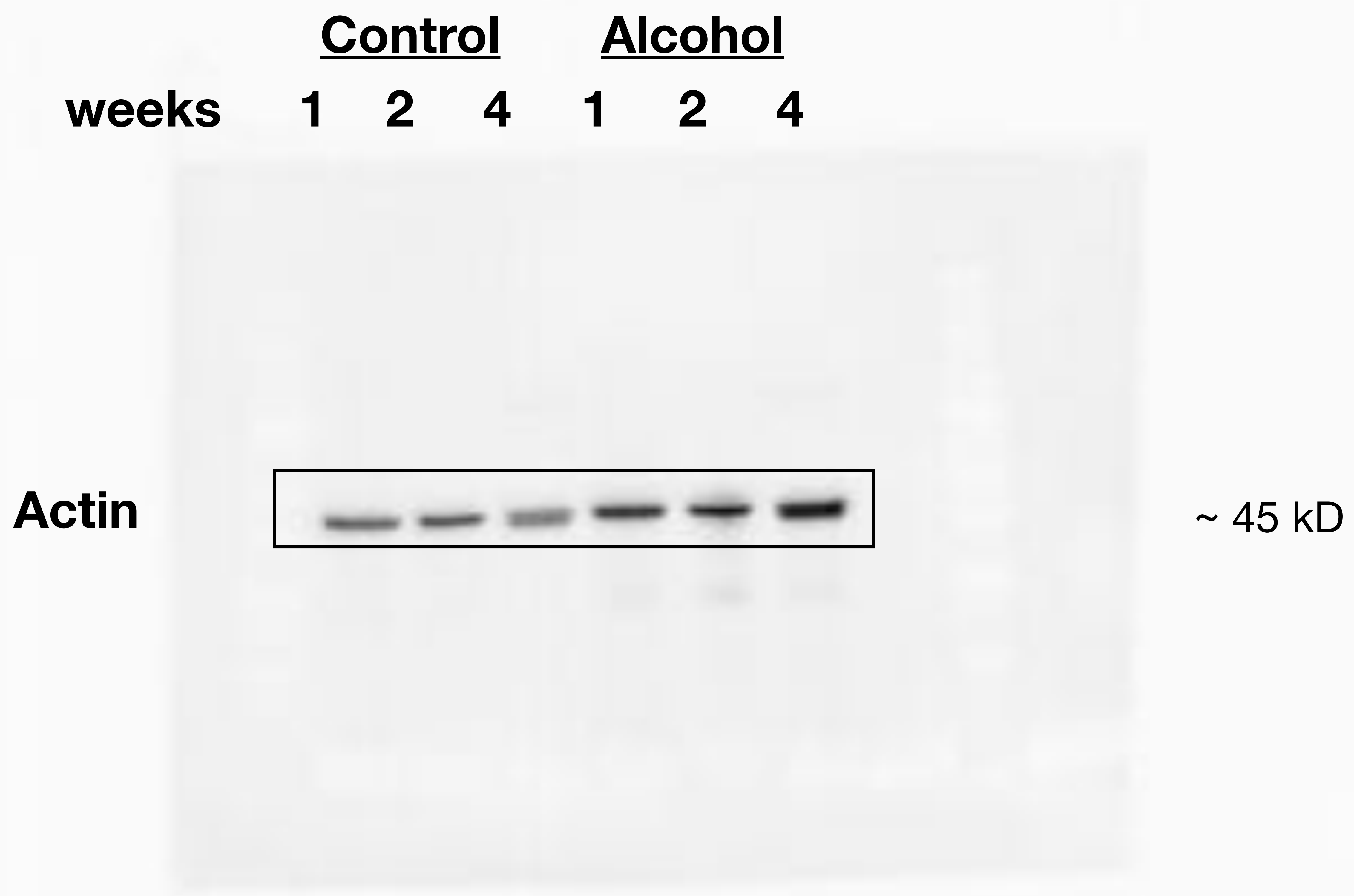

Full gel from Figure 3C

Supplementary Figure 3C

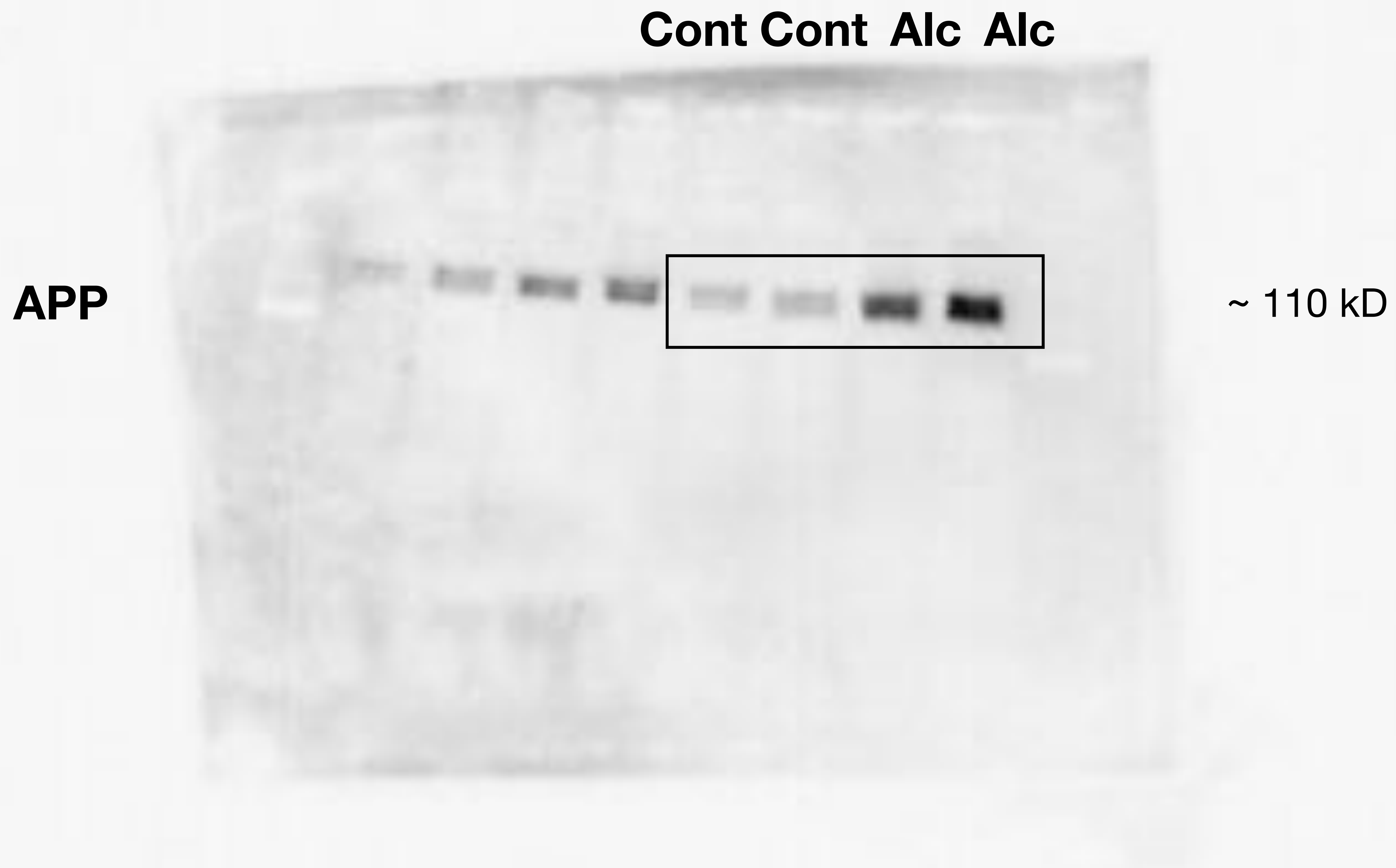

Full gel from Figure 5B

Supplementary Figure 5B

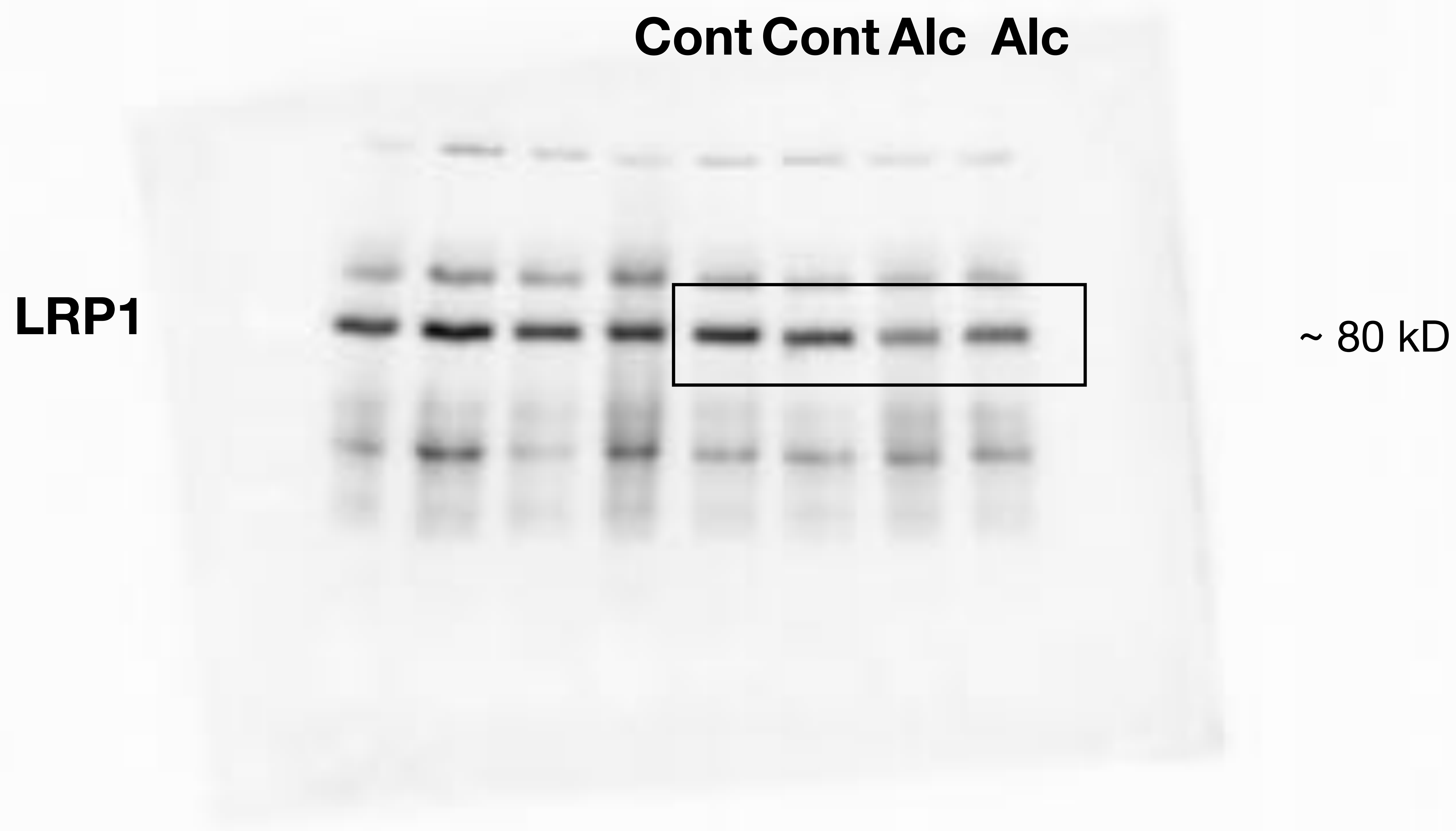

Full gel from Figure 5B

Supplementary Figure 5B

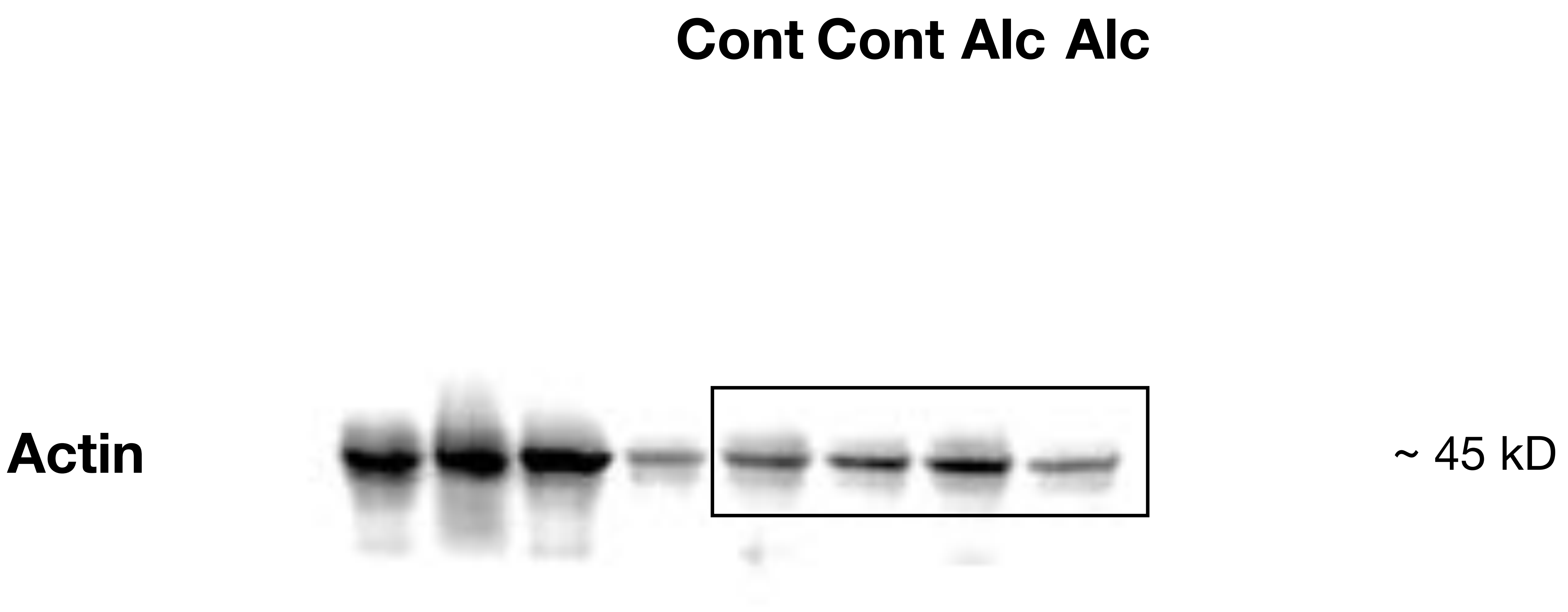

Full gel from Figure 5B

Supplementary Figure 5B

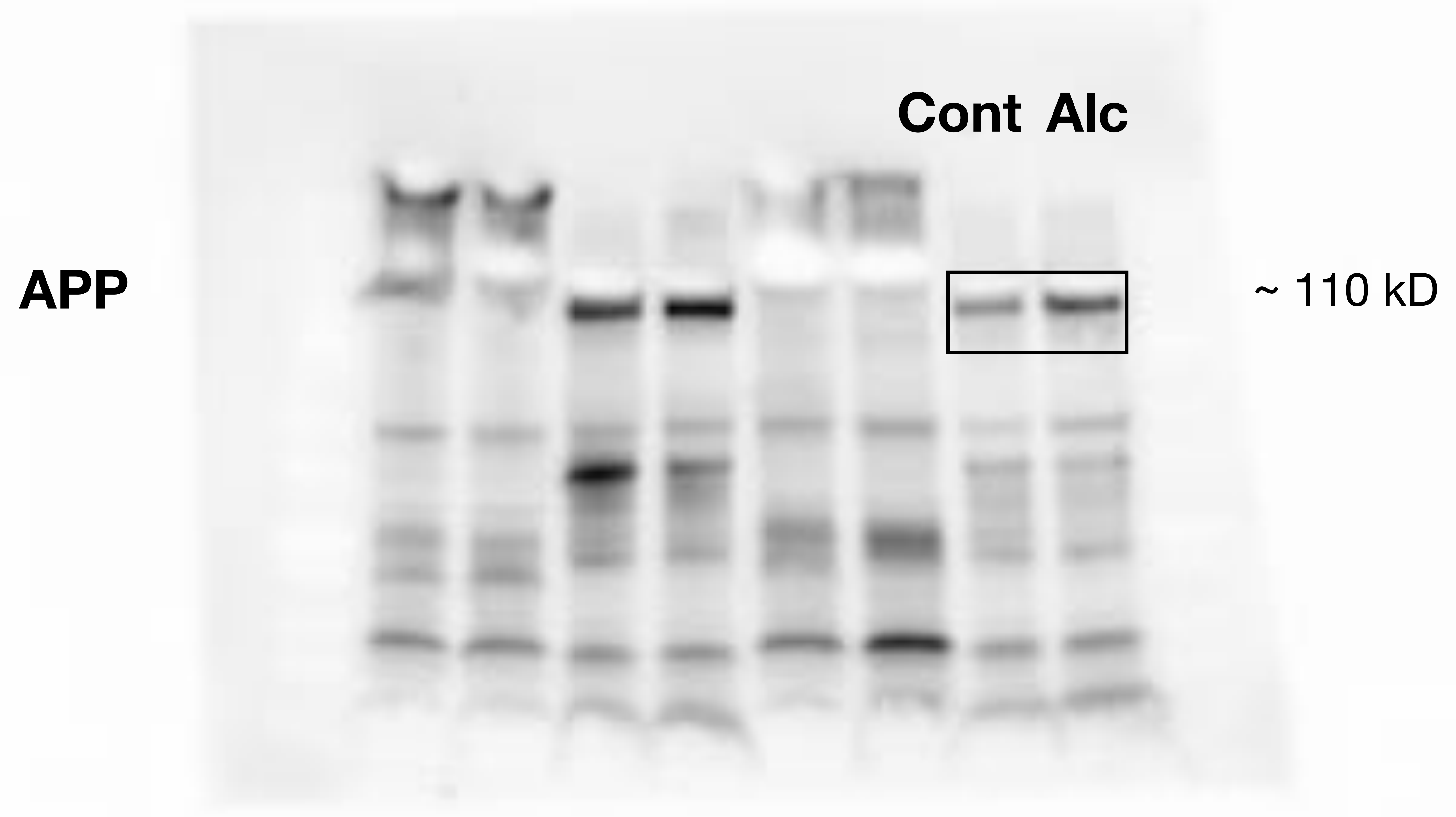

Full gel from Figure 5D

Supplementary Figure 5D

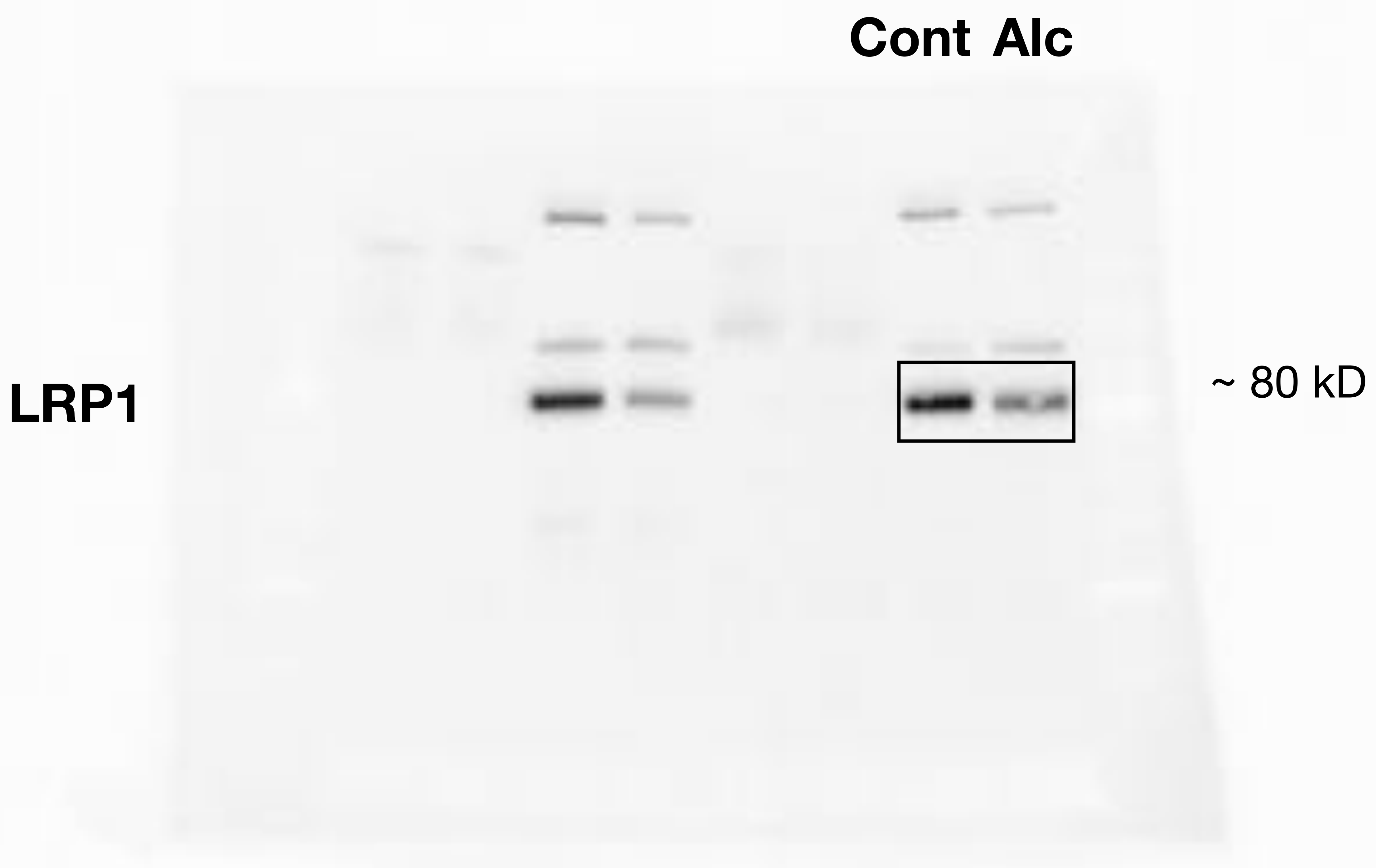

Full gel from Figure 5D

Supplementary Figure 5D

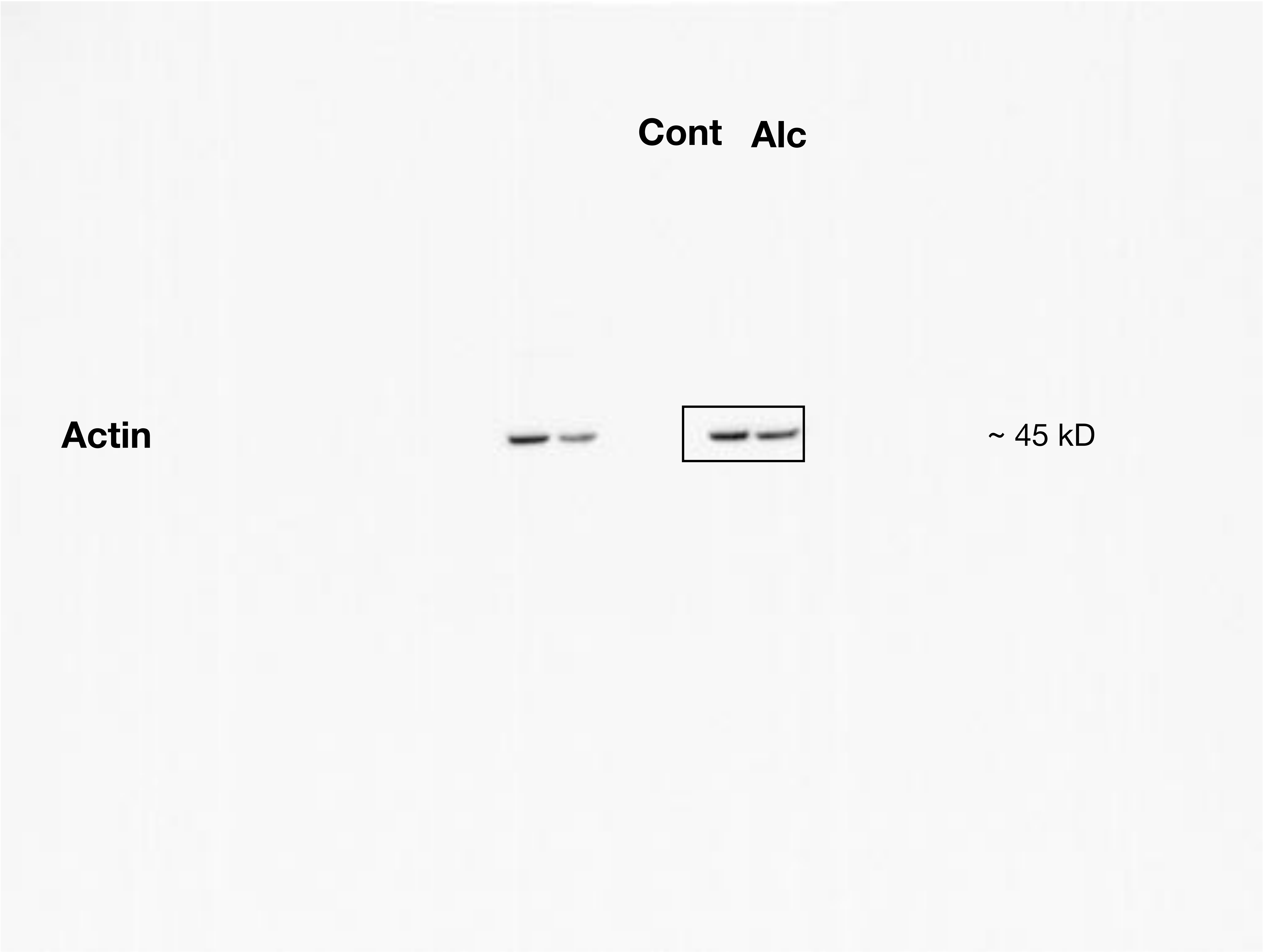

Full gel from Figure 5D

Supplementary Figure 5D

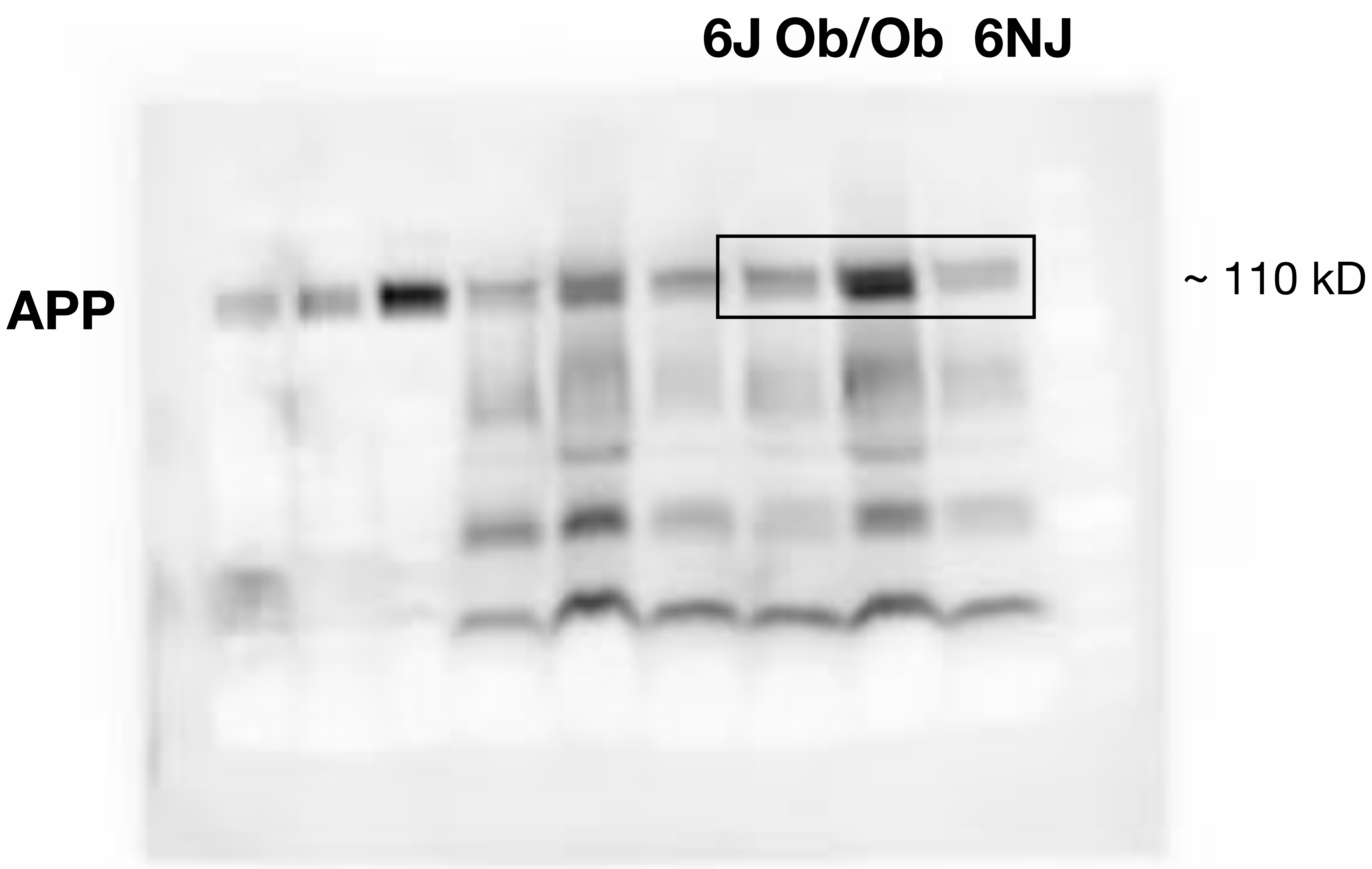

Full gel from Figure 6B

Supplementary Figure 6B

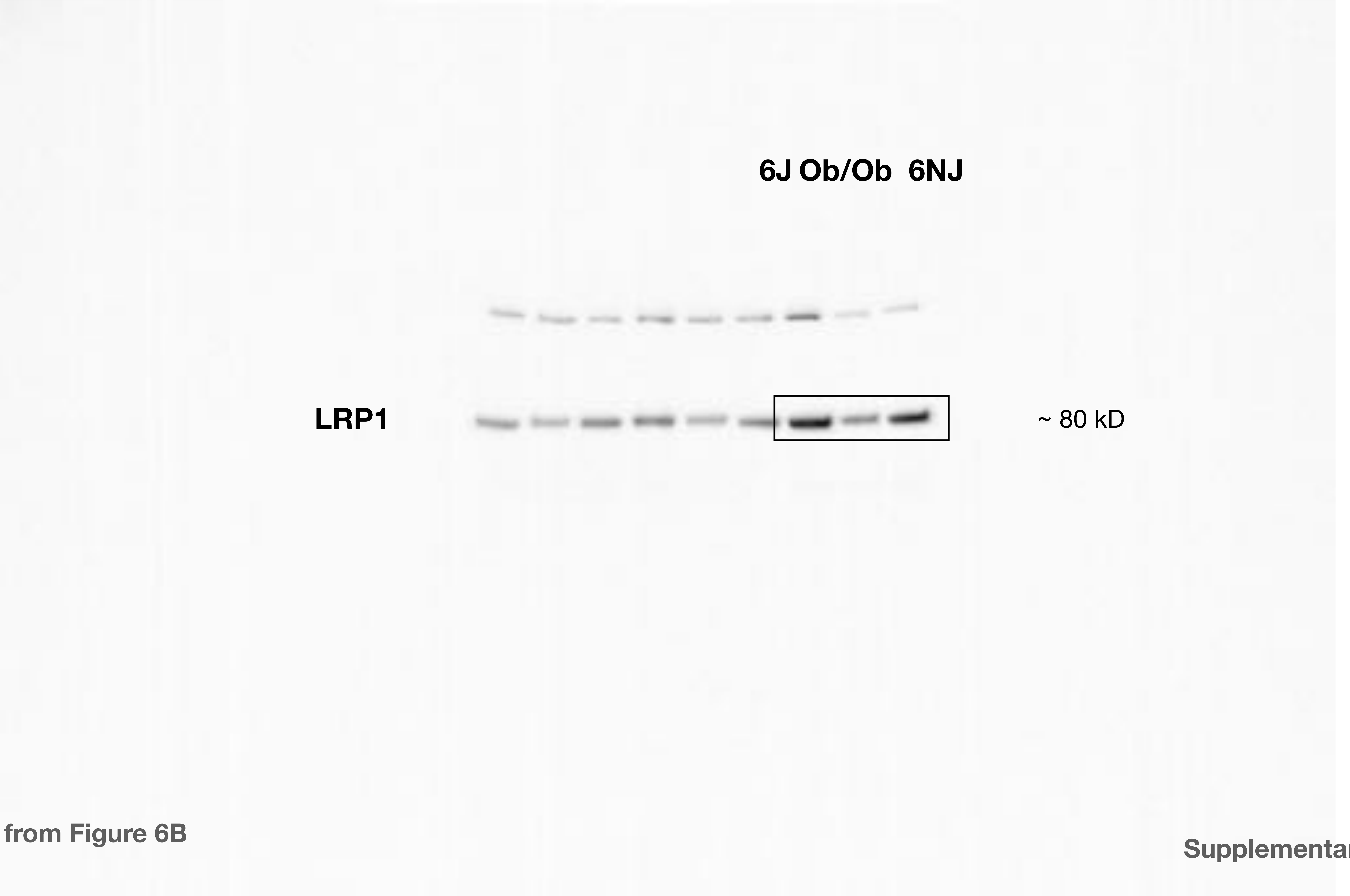

Full gel from Figure 6B

Supplementary Figure 6B

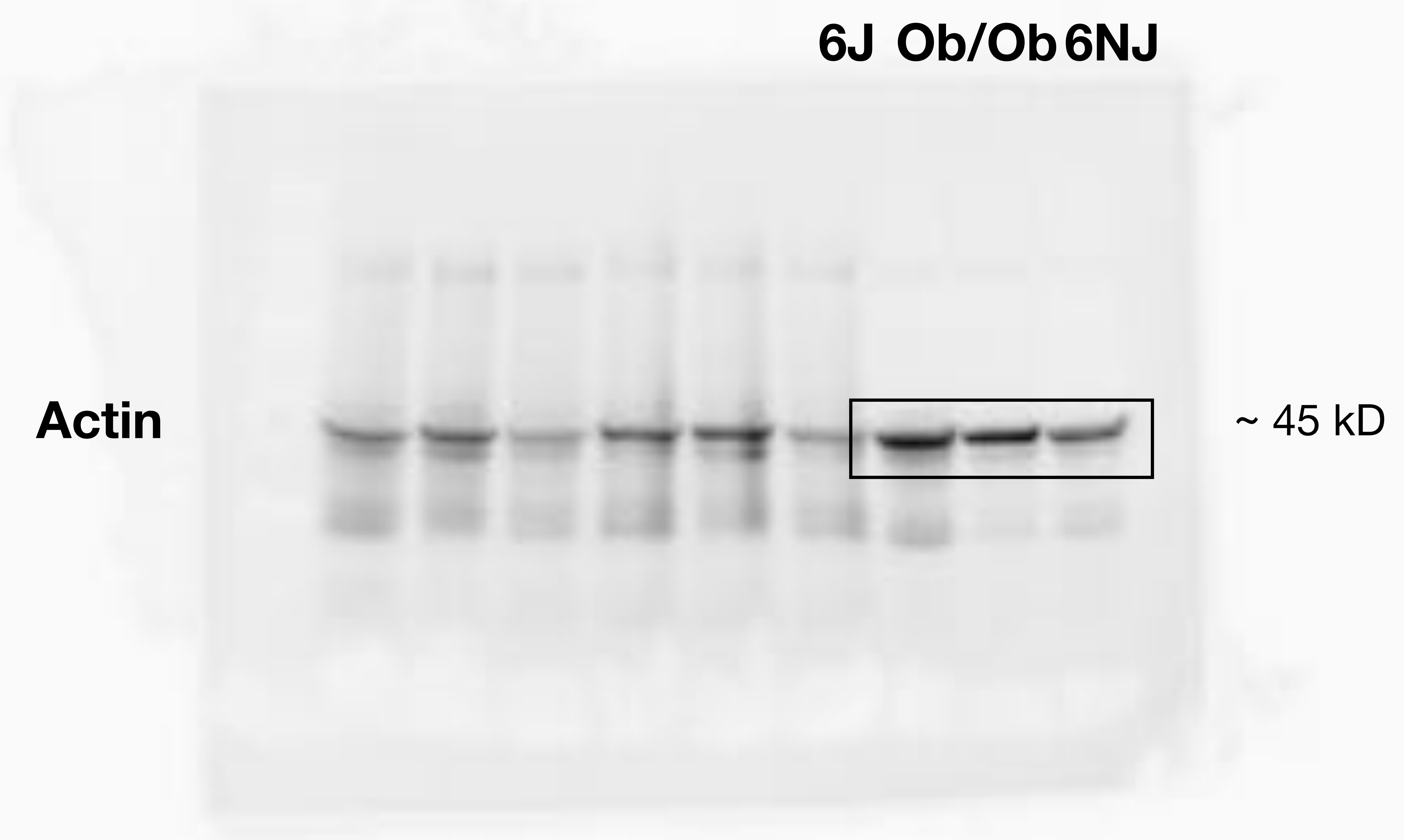

Full gel from Figure 6B

Supplementary Figure 6B
